# Accurate Digitization of the Chlorophyll Distribution of Individual Rice Leaves Using Hyperspectral Imaging and an Integrated Image Analysis Pipeline

**DOI:** 10.3389/fpls.2017.01238

**Published:** 2017-07-25

**Authors:** Hui Feng, Guoxing Chen, Lizhong Xiong, Qian Liu, Wanneng Yang

**Affiliations:** ^1^National Key Laboratory of Crop Genetic Improvement, National Center of Plant Gene Research, Agricultural Bioinformatics Key Laboratory of Hubei Province, and College of Engineering, Huazhong Agricultural University Wuhan, China; ^2^Britton Chance Center for Biomedical Photonics, Wuhan National Laboratory for Optoelectronics, and Key Laboratory of Ministry of Education for Biomedical Photonics, Department of Biomedical Engineering, Huazhong University of Science and Technology Wuhan, China

**Keywords:** chlorophyll, hyperspectral imaging, image analysis pipeline, rice, phenomics

## Abstract

Pigments absorb light, transform it into energy, and provide reaction sites for photosynthesis; thus, the quantification of pigment distribution is vital to plant research. Traditional methods for the quantification of pigments are time-consuming and not suitable for the high-throughput digitization of rice pigment distribution. In this study, using a hyperspectral imaging system, we developed an integrated image analysis pipeline for automatically processing enormous amounts of hyperspectral data. We also built models for accurately quantifying 4 pigments (chlorophyll a, chlorophyll b, total chlorophyll and carotenoid) from rice leaves and determined the important bands (700-760 *nm*) associated with these pigments. At the tillering stage, the *R*^2^ values and mean absolute percentage errors of the models were 0.827–0.928 and 6.94–12.84%, respectively. The hyperspectral data and these models can be combined for digitizing the distribution of the chlorophyll with high resolution (0.11 *mm/pixel*). In summary, the integrated hyperspectral image analysis pipeline and selected models can be used to quantify the chlorophyll distribution in rice leaves. The use of this technique will benefit rice functional genomics and rice breeding.

## Introduction

Rice is a staple food for a majority of the world population (Zhang, 2007). To meet the increasing demand due to natural disasters, human factors and the increasing world population on rice growth and yield, it is important to breed new rice varieties. In breeding research, the plant phenotype is essential for the evaluation of breeding results and gene functional analysis (Yang et al., 2013; Jasinski et al., 2016; Montagnoli et al., 2016; Negi et al., 2016). Plants contain pigments such as chlorophylls and carotenoids, which absorb light and provide energy for photosynthesis (Blackburn, 1998b). Chlorophyll is the major nitrogenous substance in higher plants and can be used for measuring plant growth (Kochubey and Kazantsev, 2007; Xue and Yang, 2009). The amount of chlorophyll present also determines a plant's photosynthetic capability, productivity and yield potential (Carter, 1998; Xue and Yang, 2009). Thus, quantification of these pigments is vital for rice phenomics and rice research.

Traditional methods for the quantification of plant pigments, including spectrophotometry (Ergun et al., 2004), paper chromatography (Sporer et al., 1954), thin-layer chromatography (Sievers and Hynninen, 1977), and high-performance liquid chromatography (Yuan et al., 1997), are time-consuming, destructive and not suitable for high-throughput phenotyping. Plant pigments have different absorption peaks under different wavelengths, which means that their spectral reflectance characteristics can be used for evaluating or distinguishing pigments (Benedict and Swidler, 1961; Gamon and Surfus, 1999). Using spectroscopy and a portable chlorophyll meter, several spectral indices have been identified, which can be used for predicting plant chlorophyll content non-destructively. Blackburn et al. reported that the amount of canopy chlorophyll a and b is related to the original reflectance at 676 and 810 nm (Blackburn, 1998a; Blackburn and Pitman, 1999). Because derivatization can reduce the noise caused by illumination, soil background, and atmosphere (Collins, 1978; Baret et al., 1992), derivative spectra have also been found to be more sensitive to the chlorophyll content and more effective than the original spectral index (Le Maire et al., 2004). Moreover, spectral indices calculated by the red edge can provide a more accurate estimation of pigment content (Miller et al., 1990; Zou et al., 2011). Researchers have also found that the ratio and normalized spectral indices are closely related to the pigment content (Moss and Rock, 1991; Chappelle et al., 1992). Yi et al. used partial least square regression and found that the reflectance at 515–550 nm, 715 and 750 nm regions had high sensitivity for detecting the carotenoid contents of cotton (Yi et al., 2014). A recent study has used hyperspectral imagery to estimate the spatial variability in the chlorophyll and nitrogen content of rice, with an *R*^2^ of 0.69–0.82 (Moharana and Dutta, 2016). Researchers also used canopy reflectance to estimate the durum wheat nitrogen status, with an RMSECV of 19.3–36.3% (Thorp et al., 2017). Portable chlorophyll meters, such as CCM-200 (Chlorophyll Content Meter) and SPAD-502 (Soil and Plant Analyzer Development), are widely used for measuring the chlorophyll content; however, manually operated portable chlorophyll meters are relatively subjective, and spectroscopy techniques cannot be used to digitize the chlorophyll distribution in rice leaves. Moreover, we summarized the recent studies on chlorophyll or nitrogen quantification that used spectral techniques (Supplementary Table 1). These studies showed that few efforts have been made to handle massive amounts of hyperspectral data and automatically digitalize the chlorophyll distribution in individual rice leaves with high-resolution.

In this study, we developed an integrated image analysis pipeline that can process extremely large amounts of hyperspectral data and built models to accurately measure 4 rice leaf pigments: chlorophyll a, chlorophyll b, total chlorophyll, and carotenoid. Moreover, by combining the hyperspectral data and the selected models, the distribution of these 4 pigments can be digitized with high resolution.

## Materials and methods

### Materials and experimental design

At the tillering stage, 10 rice accessions (*BLUE STICK, Chenwan3hao, PSBRC82, Manawthukha, Guantuibaihe, Xianggu, Wumanggaonuo, La110, Diantun502, TB154E-TB-2*, and *Ajaya*) were randomly selected from 533 rice core germplasm resources, and each accession was planted in 15 pots. The 15 pots were divided into 5 nitrogen application levels with 3 replicates: 0, 50, 100% (0.1 g *nitrogen per kg soil*), 150, and 200%. At the heading stage, 15 accessions (*RP2151-173-1-8, MR77* (*seberang*), *BASMATI 385, BLUE STICK, Chenwan3hao, PSBRC82, Manawthukha, Guantuibaihe, Xianggu, Wumanggaonuo, La110, Diantun502, TB154E-TB-2, Ajaya*, and *Bg90-2*) were randomly selected from 533 rice core germplasm resources, and 10 replicates of each accession were planted under the same nitrogen level (0.1 g *of nitrogen per kg of soil*). To test the relationship between the leaf nitrogen and hyperspectral indices, 90 accessions (seen in Supplementary Table 2) were randomly selected from 533 rice core germplasm resources and measured by an auto discrete analyzer (Smartchen 200, France), SPAD-502, and hyperspectral imaging. Detailed genetic information about these SNPs can be downloaded from the “RiceVarMap” database (http://ricevarmap.ncpgr.cn/) (Narsai et al., 2013).

### Hyperspectral imaging system and hyperspectral indices extraction

**Figure 1:**
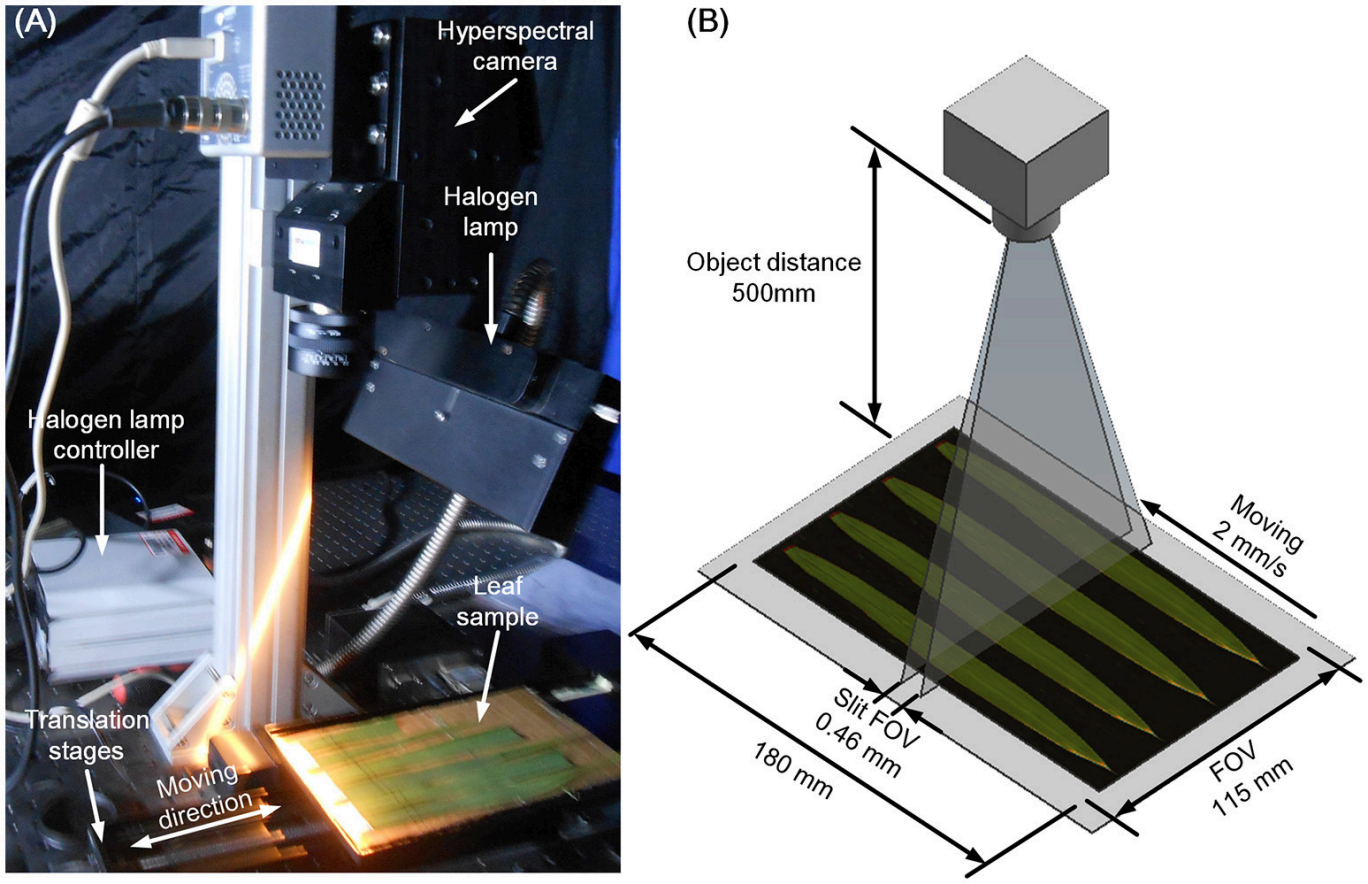
Hyperspectral imaging system **(A)** and schematic diagram **(B)**.

Three leaves were selected from the main stem of each rice plant and scanned using the hyperspectral imaging system, which consisted of 4 major parts (Figure 1A): a halogen lamp, a translation stage, a hyperspectral camera (HyperspecTM VNIR, Headwall Photonics, USA), and a computer (OXPCO3, Dell, USA). To scan three leaves of one main stem simultaneously, the field of view was set at 115 × 180 mm. The major configurations of the hyperspectral imaging system are shown in Figure 1B, and the main parameters of the hyperspectral imaging system are shown in Table 1. The data were continuously stored as a binary data stream to acquire and store the original hyperspectral data as rapidly as possible. For each sample, the data size was 1.15 GBit.

**Table 1:** Main parameters of the hyperspectral imaging system.

**Parameter**	**Value**
Object distance	500 mm
Field of view (FOV)	115 × 180 mm
Slit FOV	0.46 mm
Scan speed	2 mm/s
Hyperspectral data of one plant	1.15 GBit
Frame number	1,637
Spectral resolution	3.2 nm
Spatial resolution	0.11 mm
Spectral range	400–1,000 nm
Focal length	35 mm
Band numbers	188
CCD resolution	1,004 × 1,002

**Figure 2:**
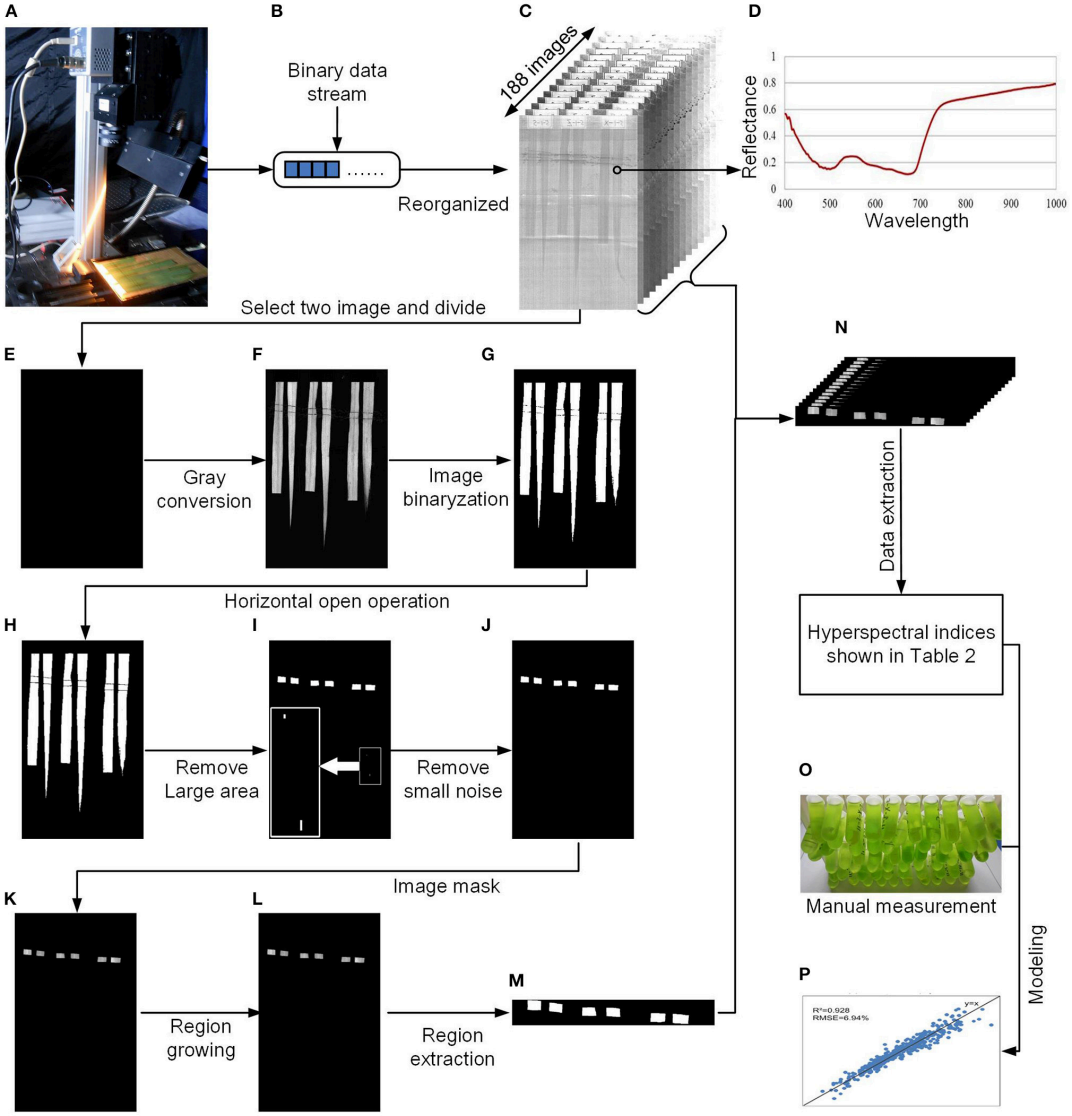
Flow chart of data processing. **(A)** System diagram. **(B)** Binary data stream acquired by the hyperspectral imaging system. **(C)** Hyperspectral images reorganized from the binary data stream. **(D)** Reflectance of the rice leaf. **(E)** Divided results (float image) of the two hyperspectral images. **(F)** Conversion of the float image to 8-bit grayscale. **(G)** Image binarization. **(H)** Horizontal open operation. **(I)** Removal of the large area. **(J)** Removal of small noise. **(K)** Image masking. **(L)** Region growing. **(M)** Region extraction. **(N)** All extractive images. **(O)** Manual measurement of the pigments. **(P)** Modeling and validation.

**Figure 3:**
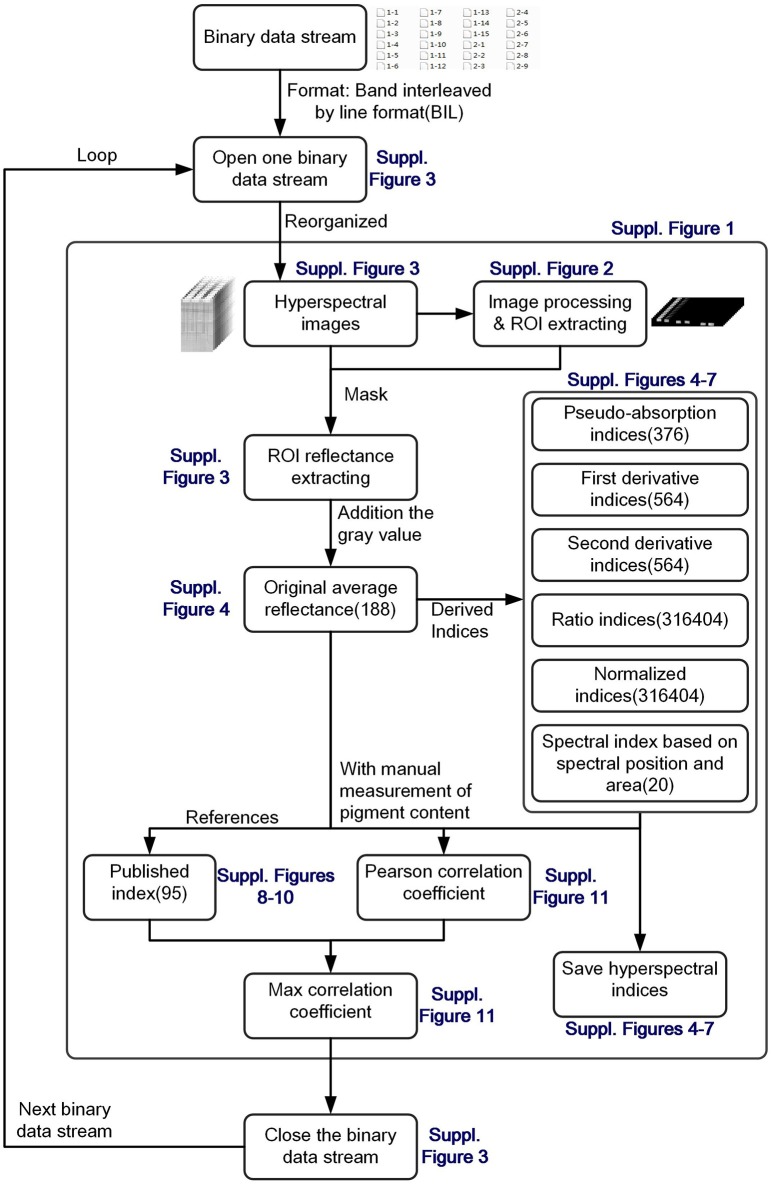
Flow chart of integrated hyperspectral image analysis pipeline.

After data acquisition, the binary data stream was reorganized to build 188 hyperspectral images under different wavelengths (Figures 2A–C). To process the massive number of images automatically, an integrated hyperspectral image analysis pipeline was developed (Figure 3). The detailed image analysis pipeline designed by LabVIEW is shown in Supplementary Figures 1–11, which included the following steps: (1) Open one binary data stream with the band interleaved by line format: The size of the hyperspectral data cube was 188 × 1,004 (*W*) × 1,637 (*H*). (2) The binary data stream was reorganized to build 188 hyperspectral images. (3) Image processing and ROI extracting: After image division, gray conversion, image binarization, horizontal open operation, removal of large areas, removal of noise, region growing, and extraction of the area of interest, a region of interest (ROI) was extracted for each leaf (Figures 2E–N). (4) ROI reflectance extracting: 188 original average reflectance indices (*R*) were obtained. (5) Derived indices extracting: These included 376 pseudo-absorption indices, 564 first derivative indices, 564 second derivative indices, 316,404 ratio indices, 316,404 normalized indices, 20 spectral indices, and 95 published indices. Finally, for each sample, 634,615 hyperspectral indices (in Table 2, among them, 20 spectral indices were shown in Supplementary Table 5, 95 published indices were shown in Supplementary Table 6) were saved. (6) Pearson's correlation coefficient was calculated, and the max correlation coefficient was obtained. (7) The binary data stream was closed.

**Table 2:** Table 2 Definition and calculation formulas of 634,615 hyperspectral indices.

**Hyperspectral index**	**Number**	Symbol^*^
Original average reflectance	188	*R*_*i*_
Pseudo-absorption index	376	lg(*R*_*i*_), lg(1/*R*_*i*_)
First derivative index	564	*d*(*R*_*i*_), *d*(lg(*R*_*i*_)), *d*(lg(1/*R*_*i*_))
Second derivative index	564	*dd*(*R*_*i*_), *dd*(lg(*R*_*i*_)), *dd*(lg(1/*R*_*i*_))
Ratio index	316404	$\frac{R_{i}}{R_{j}}$, $\frac{\mathrm{lg}\left(R_{i}\right)}{\mathrm{lg}\left(R_{j}\right)}$, $\frac{\mathrm{lg}\left(1/R_{i}\right)}{\mathrm{lg}\left(1/R_{j}\right)}$, $\frac{d\left(R_{i}\right)}{d\left(R_{j}\right)}$, $\frac{d\left(\mathrm{lg}\left(R_{i}\right)\right)}{d\left(\mathrm{lg}\left(R_{j}\right)\right)}$, $\frac{d\left(\mathrm{lg}\left(1/R_{i}\right)\right)}{d\left(\mathrm{lg}\left(1/R_{j}\right)\right)}$, $\frac{dd\left(R_{i}\right)}{dd\left(R_{j}\right)}$, $\frac{dd\left(\mathrm{lg}\left(R_{i}\right)\right)}{dd\left(\mathrm{lg}\left(R_{j}\right)\right)}$, $\frac{dd\left(\mathrm{lg}\left(1/R_{i}\right)\right)}{dd\left(\mathrm{lg}\left(1/R_{j}\right)\right)}$
Normalized index	316404	$\frac{R_{i}-R_{j}}{R_{i}+R_{j}}$, $\frac{\mathrm{lg}\left(R_{i}\right)-\mathrm{lg}\left(R_{j}\right)}{\mathrm{lg}\left(R_{i}\right)+\mathrm{lg}\left(R_{j}\right)}$, $\frac{\mathrm{lg}\left(1/R_{i}\right)-\mathrm{lg}\left(1/R_{j}\right)}{\mathrm{lg}\left(1/R_{i}\right)+\mathrm{lg}\left(1/R_{j}\right)}$, $\frac{d\left(R_{i}\right)-d\left(R_{j}\right)}{d\left(R_{i}\right)+d\left(R_{j}\right)}$, $\frac{d\left(\mathrm{lg}\left(R_{i}\right)\right)-d\left(\mathrm{lg}\left(R_{j}\right)\right)}{d\left(\mathrm{lg}\left(R_{i}\right)\right)+d\left(\mathrm{lg}\left(R_{j}\right)\right)}$, $\frac{d\left(\mathrm{lg}\left(1/R_{i}\right)\right)-d\left(\mathrm{lg}\left(1/R_{j}\right)\right)}{d\left(\mathrm{lg}\left(1/R_{i}\right)\right)+d\left(\mathrm{lg}\left(1/R_{j}\right)\right)}$, $\frac{dd\left(R_{i}\right)-dd\left(R_{j}\right)}{dd\left(R_{i}\right)+dd\left(R_{j}\right)}$, $\frac{dd\left(\mathrm{lg}\left(R_{i}\right)\right)-dd\left(\mathrm{lg}\left(R_{j}\right)\right)}{dd\left(\mathrm{lg}\left(R_{i}\right)\right)+dd\left(\mathrm{lg}\left(R_{j}\right)\right)}$, $\frac{dd\left(\mathrm{lg}\left(1/R_{i}\right)\right)-dd\left(\mathrm{lg}\left(1/R_{j}\right)\right)}{dd\left(\mathrm{lg}\left(1/R_{i}\right)\right)+dd\left(\mathrm{lg}\left(1/R_{j}\right)\right)}$
Spectral index based on spectral position and area	20	Supplementary Table 5
Published index	95	Supplementary Table 6

* *0 ≤ i ≤ 187, 0 ≤ j ≤ 187*.

### Manual measurement

After hyperspectral imaging system acquisition, the ROI of each leaf was immersed in a 95% ethanol solution. When all of the pigments had been dissolved, a spectrophotometer (L3, INESA, China) was used to measure the absorbance values of the solution at different wavelengths (470, 649, and 665 nm, Figure 2O). Finally, the contents of 4 pigments, chlorophyll a, chlorophyll b, carotenoid, and total chlorophyll, were calculated according to Equations (1)–(4) (Arnon, 1949).

(1) $$C_{a}=13.95A_{665}-6.88A_{649}$$

(2) $$C_{b}=24.96A_{649}-7.32A_{665}$$

(3) $$C_{xc}=\frac{1000A_{470}-2.05C_{a}-114.8C_{b}}{245}$$

(4) $$C=C_{a}+C_{b}$$

*C*_*a*_ is the chlorophyll a content, *C*_*b*_ is the chlorophyll b content, *C*_*xc*_ is the carotenoid content, and *C* is the total chlorophyll content. *A*_665_, *A*_649_, and *A*_470_ represent the absorbance at 665, 649, and 470 nm, respectively.

The distribution of the pigments at the two stages of plant growth is shown in Supplementary Table 3. For instance, at the tillering stage, the chlorophyll a content ranged from 61.24 to 573.63 mg/m^2^. The average value, the standard deviation, and the variable coefficient were 294.35 mg/m^2^, 92.19 mg/m^2^, and 31.32%, respectively. The correlation coefficients (*r*) between the pigments for the two stages were all above 0.88 (Supplementary Table 4), demonstrating that the concentrations of the various pigments were highly correlated.

### Data analysis and modeling

To determine the specific bands that are highly correlated with chlorophyll a, we calculated all of the correlation coefficients between 634,615 spectral indices and 4 pigments. The calculation of correlation coefficients was programmed using LabVIEW 8.6 (National Instruments, Inc., USA). The hot bands were found using the heat maps of the correlation coefficients, which were drawn using HemI software (Deng et al., 2014). After all of the indices were obtained, the best index with the highest *r* was identified and used to build 5 models (linear, power, exponential, logarithmic, and quadratic models). The statistical analyses of the 5 models (linear, power, exponential, logarithm, and quadratic model) for 4 pigments and cross-validation were implemented with LabVIEW 8.6 (National Instruments, Inc., USA). To evaluate the model performance with primary indices or multiple variables, stepwise regression analysis (SRA) was conducted using SPSS software (Statistical Product and Service Solutions, Version 13.0, SPSS Inc., USA) (Figure 2P). Finally, the digitization of pigment distribution was performed using LabVIEW 8.6 (National Instruments, Inc., USA).

## Results and discussion

### The relationship between chlorophyll a concentration and hyperspectral indices

**Figure 4:**
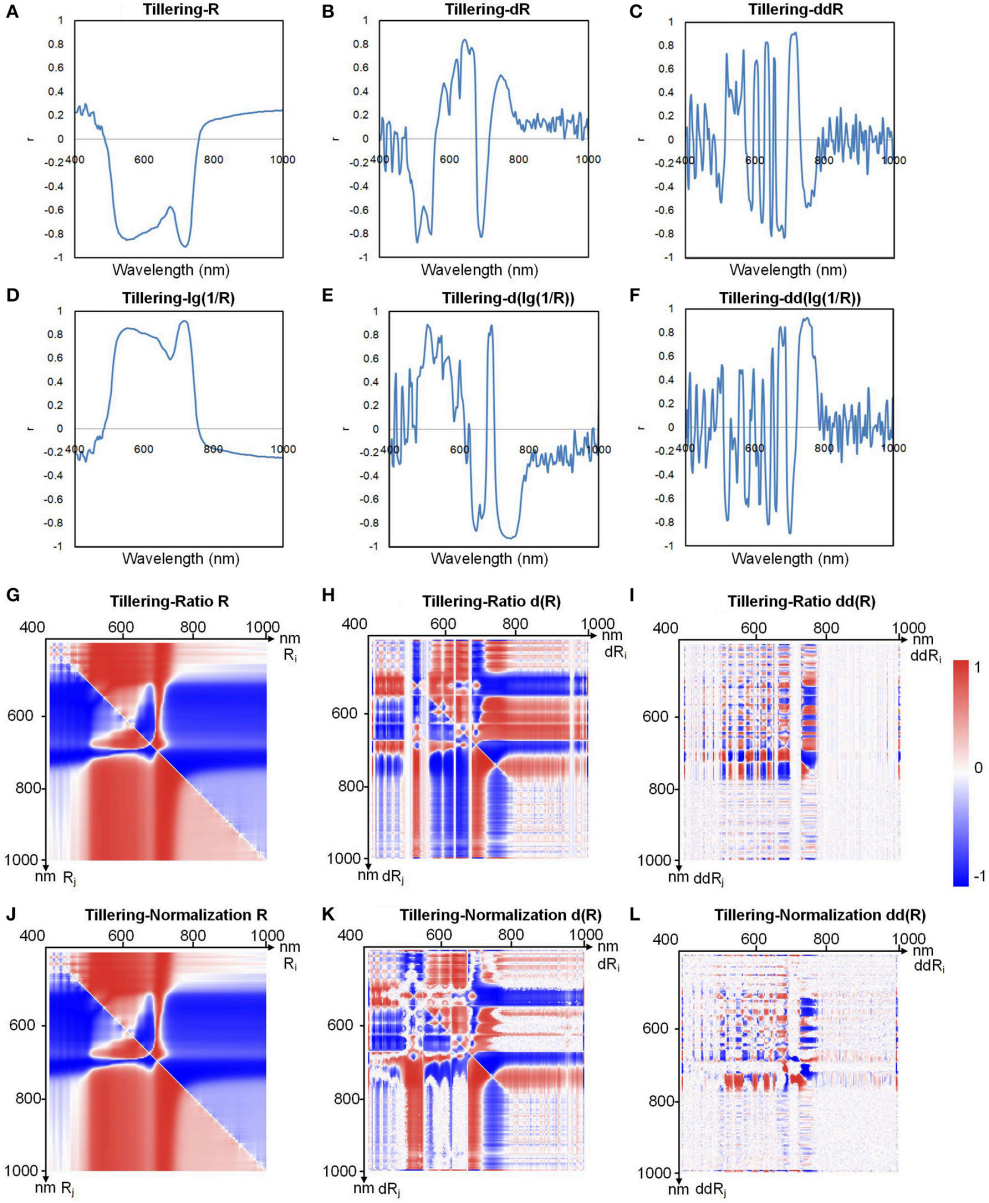
Correlation coefficients between chlorophyll a and **(A)** R, **(B)** dR, **(C)** ddR, **(D)** lg(1/R), **(E)** d(lg(1/R)), **(F)** dd(lg(1/R)), **(G)** ratio R, **(H)** ratio dR, **(I)** ratio ddR, **(J)** normalization R, **(K)** normalization dR, and **(L)** normalization ddR at the tillering stage.

The number of total indices was too large to handle (634,615 indices for each sample); thus, to decrease the number of redundant indices, we first determined the relationship between the chlorophyll content and all the hyperspectral indices. Because the pigments were highly correlated with each other (Supplementary Table 4), we used chlorophyll a as an example to define the relationship between the pigments and the hyperspectral indices. In the 500–700 nm region (Figure 4A), the reflectance *R* was inversely correlated with the chlorophyll a content, indicating that the higher the reflectance was, the lower the chlorophyll a content was. This occurred because leaves with high chlorophyll content absorbed more light, causing the reflectance to decrease (Figure 2D). From Figures 4A–F, we found that compared with a logarithmic transformation, the use of derivative transformations such as *dR, ddR, d*(*lg*(*1/R*)), and *dd*(*lg*(*1/R*)) could provide more abundant hyperspectral information.

Figures 4G–I show the correlation between the ratio index as defined in Table 2 and chlorophyll a, and Figures 4J–L show the correlation between the normalized index (also defined in Table 2) and chlorophyll a. Each point on the heat map represents the correlation coefficient between a hyperspectral index and the chlorophyll a level. The correlation coefficients for other indices and the chlorophyll a level are shown in Supplementary Figures 12, 13. When *R*_*i*_ and *R*_*j*_ were both in the 500–750 nm region, the correlation coefficient was high, sometimes even close to 1. Thus, we can infer that useful information for estimating chlorophyll a can be obtained in the wavelength range 500–750 nm.

**Figure 5:**
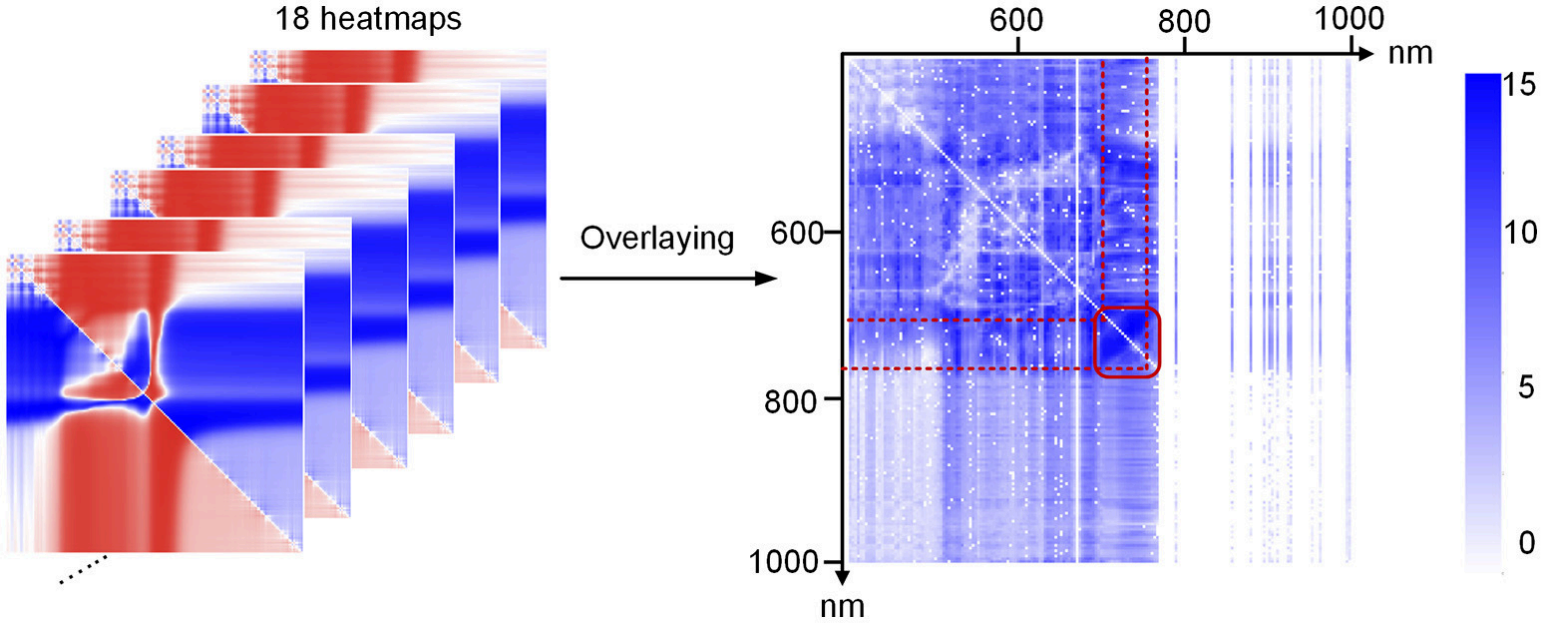
Summed coefficient image of all of the ratio and normalization coefficient images.

By comparing the data shown in Figures 4G–I, we found that for the ratio indices, the correlation between the derivative indices and chlorophyll a decreased, and the original hyperspectral index (average reflectance, *R*) showed better correlation with chlorophylla. As illustrated in Figures 4J–L, the same results could be obtained for the normalized indices. Thus, to decrease the redundant indices, primary indices, including the original average reflectance (*R*_*i*_), first derivative index (*d*(*R*_*i*_)), second derivative index (*dd*(*R*_*i*_)), ratio index (*R*_*i*_*/R*_*j*_), and normalized index ((*R*_*i*_*-R*_*j*_)*/*(*R*_*i*_+*R*_*j*_)), were used for the subsequent modeling and prediction of chlorophyll levels. A combined heat map obtained by adding together all of the heat maps of ratio and normalization coefficients is shown in Figure 5. From this, we found that the region of the highest correlation was located between 700 and 760 nm. If only the primary indices in the 700–760 nm region were used, the number of indices would decrease from 634, 615 to 483.

### Linear modeling with a single variable

**Figure 6:**
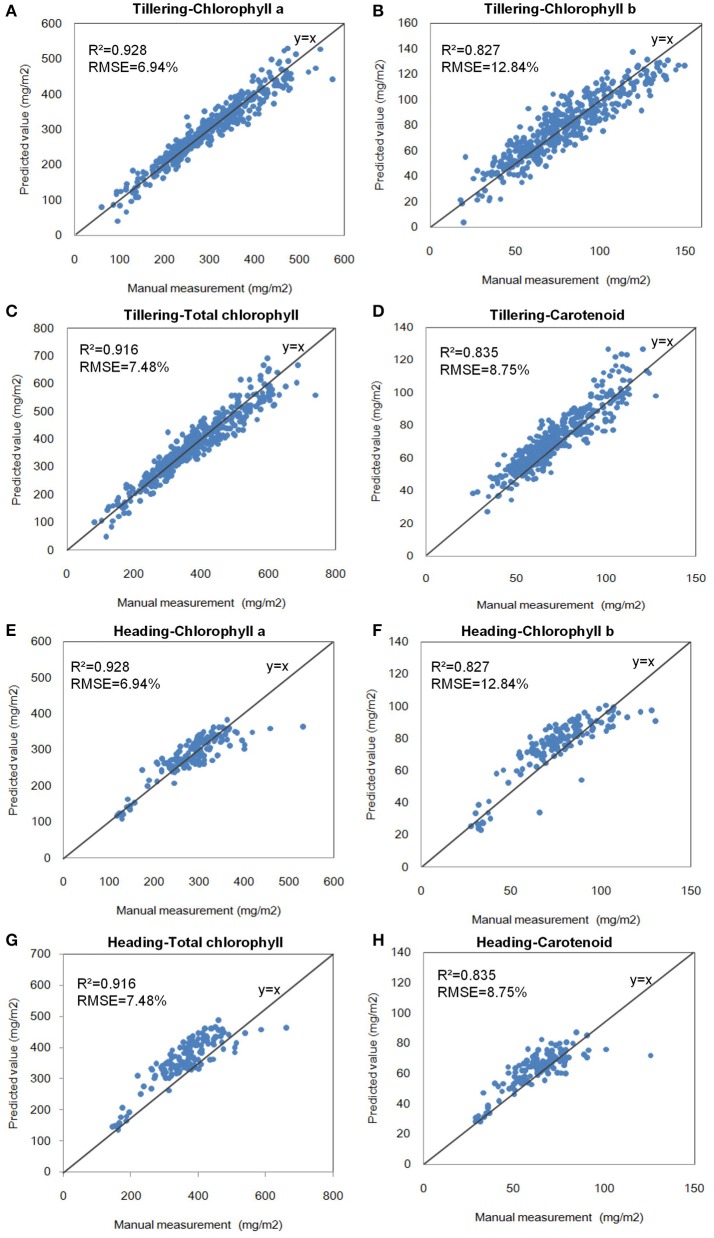
Scatter plots of the single-variable model of pigments at the two stages. **(A)** Chlorophyll a at the tillering stage. **(B)** Chlorophyll b at the tillering stage. **(C)** Total chlorophyll at the tillering stage. **(D)** Carotenoid at the tillering stage. **(E)** Chlorophyll a at the heading stage. **(F)** Chlorophyll b at the heading stage. **(G)** Total chlorophyll at the heading stage. **(H)** Carotenoid at the heading stage.

After all of the indices were calculated, the hyperspectral indices with the highest correlation coefficients (*r*) of the pigments were selected for the modeling step, as shown in Table 3. The single-variable model for 4 pigments at the tillering and heading stages is shown in Table 4, which show that *R*^2^ ranged from 0.654 to 0.928, and the mean absolute percentage error (MAPE) was 6.94–12.84%. The scatter plots and the distribution of the relative error are shown in Figure 6 and Supplementary Figure 14, respectively, which show the points to be evenly distributed around the line y = x and that most of the relative error within the range ±10%. A 5-fold cross-validation of the single variable model for the 4 pigments at the two stages is shown in Table 4, which shows the ranges of *R*^2^ and MAPE as 0.671–0.930 and 7.49–13.02%, respectively.

**Table 3:** Hyperspectral indices that displayed the highest *r* values selected from all indices or primary indices for the 4 pigments and comparison with published indices.

**Stage**	**Pigment**	**Best index selected in all indices**	***r***	**Best index selected in primary indices**	***r***	**Published indices with the highest r**	***r***
Tillering stage	Chlorophyll a	$\frac{\mathrm{lg}\left(R_{715}\right)}{\mathrm{lg}\left(R_{500}\right)}$	0.963	*R*_714_	0.919	$\frac{D_{705}}{D_{702}}$ (Zarco et al., 2002)	0.919
	Chlorophyll b	$\frac{\mathrm{lg}\left(R_{715}\right)}{\mathrm{lg}\left(R_{660}\right)}$	0.909	*R*_721_	0.886	$\frac{R_{657}}{R_{700}}$ (Chappelle et al., 1992)	0.876
	Total chlorophyll	$\frac{\mathrm{lg}\left(R_{715}\right)}{\mathrm{lg}\left(R_{500}\right)}$	0.957	*R*_718_	0.920	$\frac{R_{657}}{R_{700}}$ (Chappelle et al., 1992)	0.915
	Carotenoid	$\frac{\mathrm{lg}\left(R_{718}\right)}{\mathrm{lg}\left(R_{450}\right)}$	0.914	*dd*(*R*_724_)	0.848	$\frac{R_{657}}{R_{700}}$ (Chappelle et al., 1992)	0.852
Heading stage	Chlorophyll a	$\frac{d\left(R_{997}\right)-d\left(R_{747}\right)}{d\left(R_{997}\right)+d\left(R_{747}\right)}$	0.873	*dd*(*R*_721_)	0.836	$\frac{R_{728}-R_{434}}{R_{720}-R_{434}}$ (Le Maire et al., 2004)	0.732
	Chlorophyll b	$\frac{d\left(R_{997}\right)-d\left(R_{728}\right)}{d\left(R_{997}\right)+d\left(R_{728}\right)}$	0.855	*R*_714_	0.835	$\frac{R_{728}-R_{434}}{R_{720}-R_{434}}$ (Le Maire et al., 2004)	0.675
	Total chlorophyll	$\frac{d\left(R_{997}\right)-d\left(R_{747}\right)}{d\left(R_{997}\right)+d\left(R_{747}\right)}$	0.872	*R*_714_	0.837	$\frac{R_{728}-R_{434}}{R_{720}-R_{434}}$ (Le Maire et al., 2004)	0.726
	Carotenoid	$\frac{d\left(\mathrm{lg}\left(1/R_{747}\right)\right)-d\left(\mathrm{lg}\left(1/R_{792}\right)\right)}{d\left(\mathrm{lg}\left(1/R_{747}\right)\right)+d\left(\mathrm{lg}\left(1/R_{792}\right)\right)}$	0.809	*dd*(*R*_721_)	0.782	$\frac{R_{728}-R_{434}}{R_{720}-R_{434}}$ (Le Maire et al., 2004)	0.668

**Table 4:** Details of the single-variable models for the 4 pigments.

**Stage**	**Chlorophyll**	Single-variable model^*^	***R*^2^**	**MAPE**	**RMSE (*mg/m*^2^)**	**5-fold cross validation**				
						**Modeling**			**Validation**	
						***R*^2^**	**MAPE**	**RMSE (*mg/m^2^*)**	**MAPE**	**RMSE (*mg/m^2^*)**
Tillering stage	Chlorophyll a	*y* = 1217.948*x*_1_ − 301.306	0.928	6.94%	24.73	0.930	6.93%	24.721	7.49%	24.944
	Chlorophyll b	*y* = 557.723*x*_2_ − 126.609	0.827	12.84%	11.19	0.832	12.86%	11.184	13.02%	11.323
	Total chlorophyll	*y* = 1596.104*x*_1_ − 405.674	0.916	7.48%	34.18	0.918	7.48%	34.179	7.53%	34.221
	Carotenoid	*y* = 188.087*x*_3_ − 22.582	0.835	8.75%	7.76%	0.833	8.74%	7.760	8.91%	7.786
Heading stage	Chlorophyll a	*y* = 7874.223*x*_4_ − 8009.138	0.761	8.25%	32.73	0.770	8.24%	32.703	8.46%	33.023
	Chlorophyll b	*y* = 933.651*x*_5_ − 1006.710	0.731	10.93%	10.40	0.732	10.94%	10.384	11.24%	10.594
	Total chlorophyll	*y* = 10159.684*x*_4_ − 10340.279	0.761	8.70%	42.32	0.768	8.68%	42.218	8.59%	43.702
	Carotenoid	*y* = 175.113*x*_6_ − 52.628	0.654	9.14%	8.53	0.671	9.13%	8.528	9.29%	8.586

* *x_1_ = log(R_715_)/log(R_500_), x_2_ = log(R_715_)/log(R_660_), x_3_ = log(R_718_)/log(R_450_), x_4_ = (d(R_997_)−d(R_747_))/(d(R_997_)+d(R_747_)), x_5_ = (d(R_997_)−d(R_728_))/(d(R_997_)+d(R_728_)), x_6_ = (d(lg(1/R_747_))−d(lg(1/R_792_)))/(d(lg(1/R_747_))+d(lg(1/R_792_)))*.

**Figure 7:**
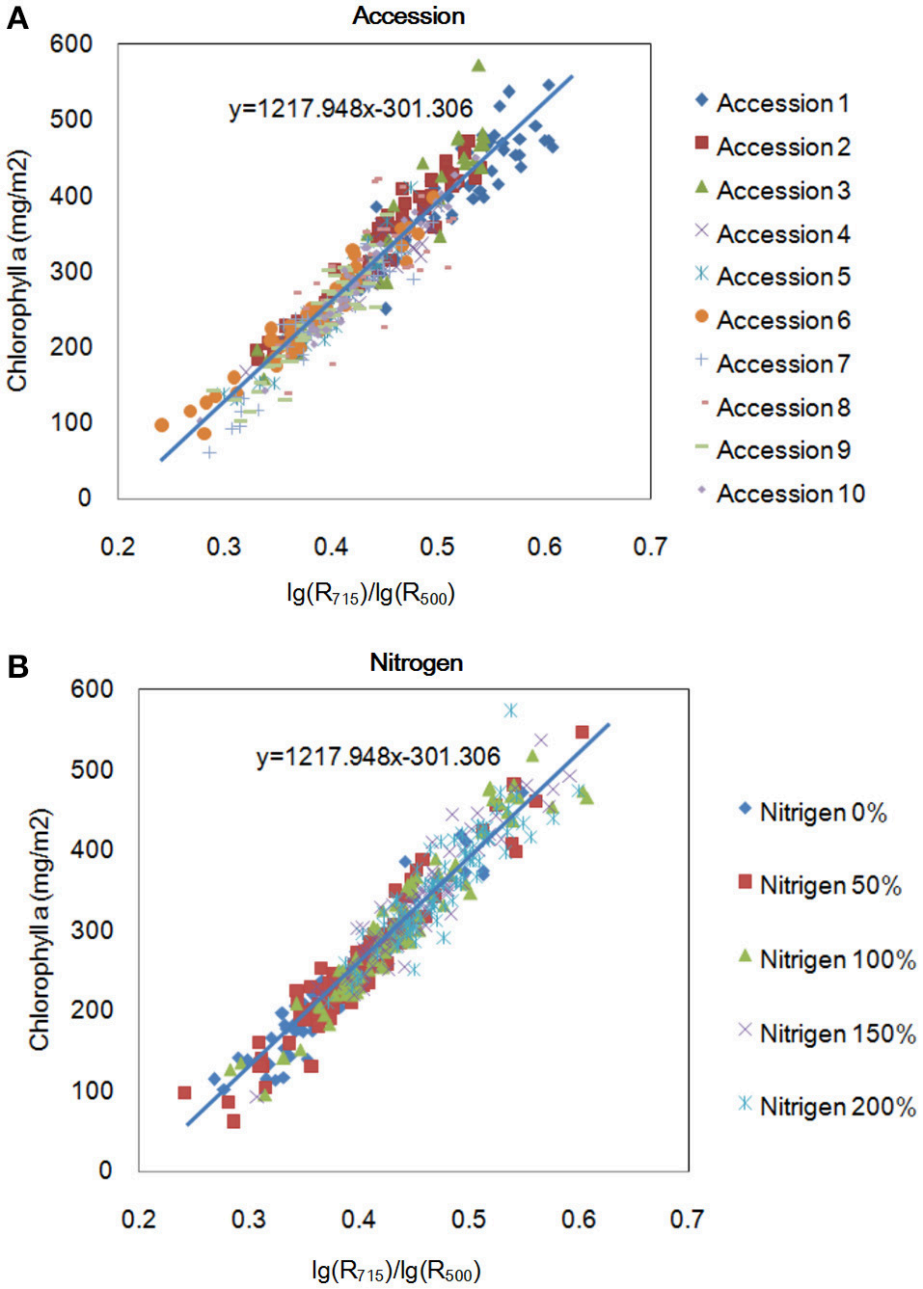
Relationship between R_715_/R_500_ and chlorophyll a content for different accessions **(A)** and for the same accessions under different nitrogen application levels **(B)** at the tillering stage.

To evaluate the model's robustness, we evaluated the relationship between *lg*(*R*_715_)*/lg*(*R*_500_) and the chlorophyll a level for different accessions grown under different nitrogen regimes at the tillering stage (Figure 7). The model was not sensitive to accession or the nitrogen application level. Figure 7B shows that the amount of chlorophyll an increased with increase in the nitrogen application level. Moreover, we also compared the best model for the 4 pigments in this study with the published indices, as shown in Table 3 and Supplementary Table 6. The correlation between the pigments and the indices selected in this study (0.81–0.96) was higher than the correlation between the pigments and the published index with the highest *r* (0.67–0.92). On the other hand, all of the published indices with high *r* values were based on at least one wavelength in the range of 700–760 nm, implying that this range (700–760 nm) is important for the quantification of leaf chlorophyll.

### Comparison of linear and non-linear models

To determine the best model for determination of chlorophyll a levels, 5 models, including the linear, power, exponential, logarithmic, and quadratic models, were compared. The results are shown in Table 5. We found that the linear model had the highest *R*^2^ (0.928) and lowest MAPE (6.94%). Based on the relative robustness of the models, the linear model was selected as the final model for the quantification of chlorophyll. The results also indicate that the best relationship between the chlorophyll content and the index value was linear in our study.

**Table 5:** Statistical summary of the 5 developed models for chlorophyll a estimation (sample size = 425)^*^.

**No**.	**Model classification**	**Model**	**Coefficients**	***R*^2^**	**MAPE**	**SD_*APE*_**
1	Linear	*y* = *a*_0_ + *a*_1_ × *x*	a_0_ = –284.78	0.928	6.94%	7.86%
			a_1_ = 1351.04			
2	Power	*y* = *a*_0_*x*^*a*^1^^	a_0_ = 1469.65	0.913	7.85%	10.16%
			a_1_ = 1.92			
3	Exponential mode	*y* = *a*_0_ × *e*^*a*^^1 × *x*^	a_0_ = 49.69	0.887	9.65%	14.24%
			a_1_ = 4.11			
4	Logarithmic	*y* = *a*_0_*ln*(*a*_1_*x*)	a_0_ = 568.05	0.911	8.23%	9.73%
			a_1_ = 3.96			
5	Quadratic	$y=a_{0}+a_{1}\times x+a_{2}\times x^{2}$	a_0_ = –305.10	0.922	7.54%	7.54%
			a_1_ = 1446.59			
			a_2_ = –109.76			

* *y is chlorophyll a, x is $\frac{\mathrm{lg}\left(R_{715}\right)}{\mathrm{lg}\left(R_{500}\right)}$*.

### Comparison of models with all indices and models with primary indices

To compare the models that use all indices with those that use primary indices, we used 634,615 indices, and 483 primary indices for evaluating the model performance. The results (Table 3) showed that the highest *r* of the models that used primary indices (0.782–0.920) was similar to the highest *r* of the models that used all indices (0.809–0.963), indicating that the models that use primary indices are sufficiently accurate for the quantification of the 4 pigments. If only the primary indices were extracted and analyzed, the volume of hyperspectral data decreased from hundreds of thousands to hundreds, which dramatically reduced the workload of data acquisition and data analysis. The results of this comparison are shown in Table 3 and Supplementary Table 7.

### Linear modeling with multi-variables

We also evaluated the model performance using multi-variables. To faciliate the evaluation, only some primary indices, including *R, dR*, and *ddR*, were used to build the model using a stepwise regression analysis. The results (Supplementary Table 8) showed that *R*^2^ and *R*_*adj*_^2^ increased slightly and that MAPE and RMSE decreased slightly as the number of independent variables increased. The distribution of the relative error of the model using a stepwise regression analysis and multi-variables for chlorophyll a at the tillering stage is shown in Supplementary Figure 15, and 5-fold cross-validation of these models is shown in Supplementary Table 8.

### Digitization of leaf chlorophyll distribution

**Figure 8:**
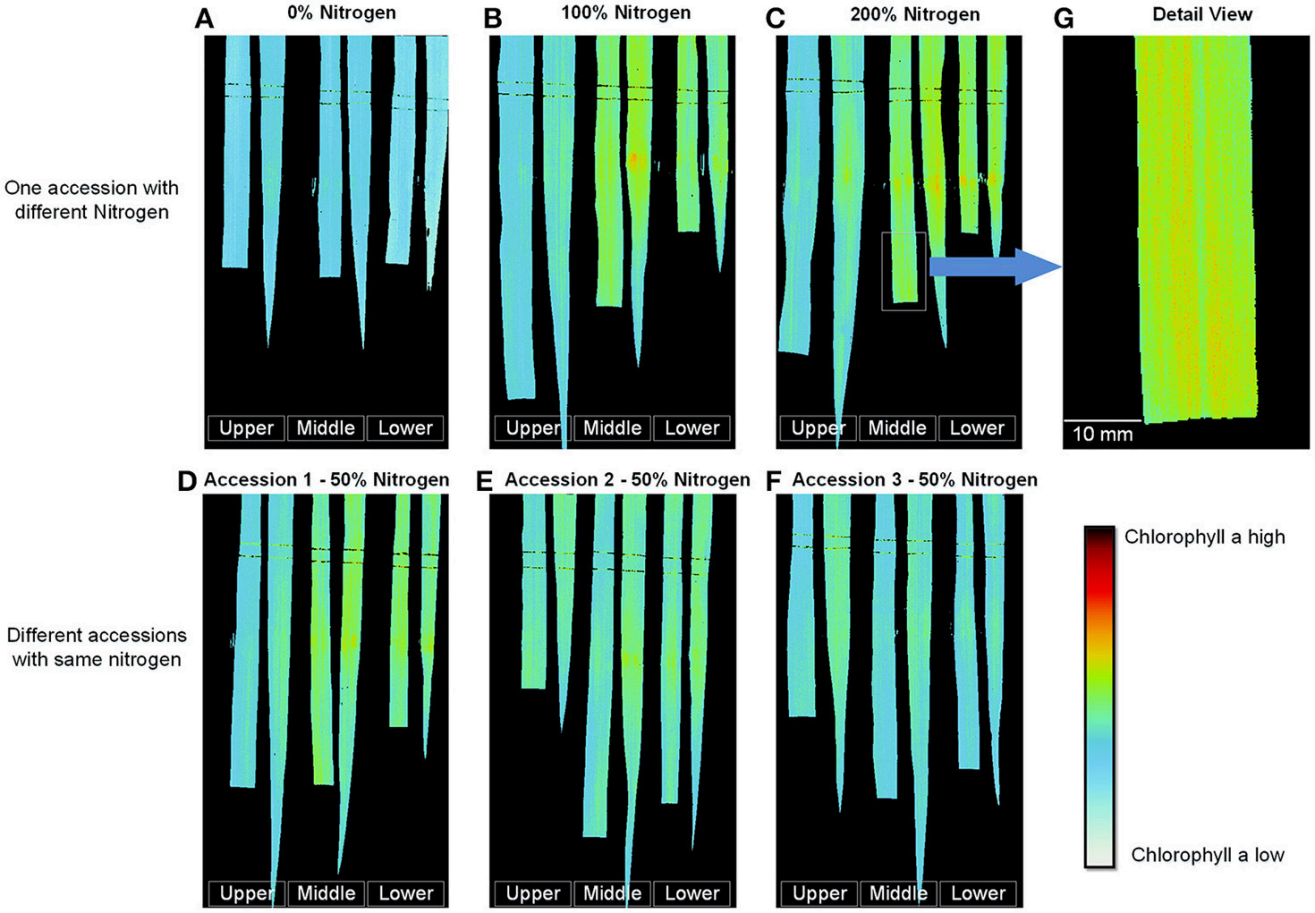
Digitization of the leaf chlorophyll distribution at the tillering stage. **(A–C)** One accession with different nitrogen application levels. **(D–F)** Different accessions with the same nitrogen application level. **(G)** Detailed image of **(C)**. (To facilitate comparison, the gray stretching parameters of **A–C** were the same, and the gray stretching parameters of **D–F** were the same).

After the best single-variable model was built, it was used to digitize the leaf chlorophyll distribution at a high resolution (0.11 mm/pixel), as shown in Figure 8 (pseudo-color images). Figures 8A–C show the results obtained for one accession grown under different nitrogen application levels; with increasing nitrogen application, the chlorophyll a content increased dramatically. The chlorophyll a content of different accessions grown under the same nitrogen application level also varied (Figures 8D–F). Figures 8A–F show that for most samples, the chlorophyll concentration in the middle portion of the leaf was the highest, followed by the lower leaf and the upper leaf. Moreover, for the same leaf, the chlorophyll a content of the leaf vein was less than that of the leaf pulp, as shown in Figure 8G.

### Modeling nitrogen with hyperspectral imaging

**Figure 9:**
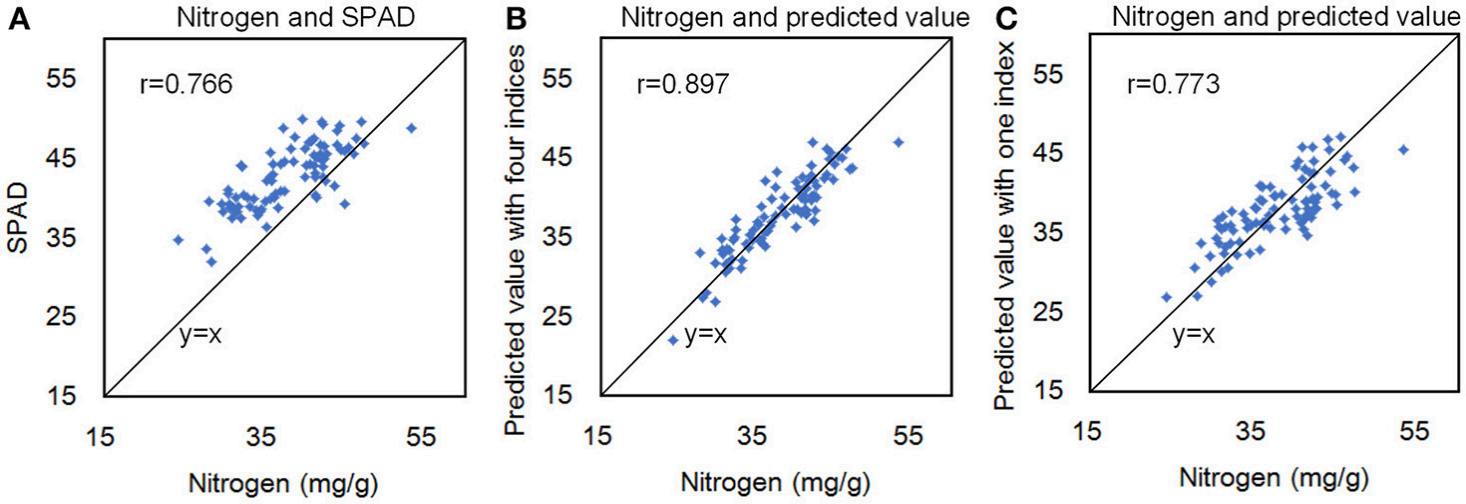
The correlation coefficient (r) between the SPAD value and the nitrogen content **(A)**, between the nitrogen content and hyperspectral measurements with 4 indices **(B)**, and between the nitrogen content and hyperspectral measurements with 1 index **(C)**.

A recent study showed that *R*^2^ between the total chlorophyll content and leaf nitrogen content of Papaya plants (Castro et al., 2011) could reach 0.78, and hyperspectral reflectance measurements could reflect the canopy nitrogen content of winter wheat (Zhou et al., 2016). To test the correlation between the nitrogen and hyperspectral indices in rice, we measured 90 rice accessions, selected from 533 rice core germplasm resources, using an auto discrete analyzer (Smartchen 200, France), SPAD-502, and hyperspectral imaging. The correlation coefficient (*r*) between the SPAD value and the nitrogen content was 0.766 (Figure 9A), and r between the nitrogen content and hyperspectral measurements with 4 indices was 0.897 (Figure 9B). Moreover, only using one index, the *r* between the nitrogen content and hyperspectral measurements was 0.773 (Figure 9C). The results showed that nitrogen in rice plants could also be quantified using hyperspectral imaging.

### Comparison of recent related studies for quantifying chlorophyll or nitrogen distribution

We compared the present research with recent related studies and found that several key wavelengths that reflect chlorophyll, such as cotton at 715 and 750 nm (Yi et al., 2014), winter wheat at 705 nm and the red edge (Zhou et al., 2016), and grass at 690–750 (Tong and He, 2017), were co-determined. Moreover, the commonly adopted tools, such as ENVI and SAS, handled enormous amounts of hyperspectral data, particularly image analysis, with difficulty. To relieve the bottleneck, we developed an integrated image analysis pipeline in this study. With a single variable, the measuring accuracy of chlorophyll, *R*^2^, ranged from 0.654 to 0.928. Moreover, due to using hyperspectral imaging in a higher resolution (0.11 mm/pixel), the distribution of leaf chlorophyll could be clearly visualized. The goal of this article was to quantify the chlorophylls in individual rice leaves, which should be tested and verified in the field in future. Combining the current field phenotyping tools, such as field phenotyping at the plot level (Andrade-Sanchez et al., 2014) and movable imaging chambers in the field (Busemeyer et al., 2013), the integrated image analysis pipeline could be expanded to the field. Moreover, combined hyperspectral imaging with a novel sensor for structure imaging, such as a micro-CT (Mineyuki, 2014) and 3D laser scanning (Paulus et al., 2014), could also reconstruct the 3D distribution of chlorophyll in a high resolution.

## Conclusions

In this study, we used a hyperspectral imaging system to develop an integrated image analysis pipeline to handle extremely large amounts of hyperspectral data automatically. We also built models that could be used to accurately quantify 4 rice leaf pigments and identify the important spectral bands (700–760 nm) associated with these pigments. Moreover, by combining the hyperspectral data and these models, the distribution of chlorophyll could be digitized with high resolution (0.11 mm/pixel). In the future, the pipeline and selected models can potentially be applied to quantify the chlorophyll distribution in individual plants non-destructively. Evidence from related works shows that the image analysis pipeline combined with hyperspectral imaging could also be extended for co-determining wavelengths for quantifying chlorophyll in other crops.

## Author contributors

HF and WY designed the research, performed the experiments, analyzed the data and wrote the manuscript. GC provided the rice samples and also performed experiments. LX and QL supervised the project, designed the research, and wrote the manuscript.

### Conflict of interest statement

The authors declare that the research was conducted in the absence of any commercial or financial relationships that could be construed as a potential conflict of interest. The reviewer RJW and handling Editor declared their shared affiliation, and the handling Editor states that the process met the standards of a fair and objective review.

## Supplementary material

Supplementary Figure 1 Flow chart of the program. The number represents the processing module of the following Supplementary Figures 2–11.

Supplementary Figure 2 Program for image processing and ROI extraction. **(A)** Reorganized two images from the binary data stream (1), **(B)** Segment the leaf part from the background with the two images (2), **(C)** Image processing for the ROI extraction from the leaf part (3), **(D)** ROI extraction and save (4).

Supplementary Figure 3 Program for ROI reflectance extraction. **(A)** The main program for ROI reflectance extraction of all samples (5), **(B)** Creating the Excel file (6), **(C)** Savin the Excel file (7), **(D)** The sub-program for ROI reflectance extraction of single sample (8), **(E)** Applying the Supplementary Figure 2 results to the current data processing (9).

Supplementary Figure 4 Program for calculation of the original average reflectance **(A)** (10) and the spectral index based on spectral position and area **(B)** (11).

Supplementary Figure 5 Program for calculation of the first and second derivatives (12).

Supplementary Figure 6 Program for calculation of the pseudo-absorption index. **(A)** The calculation of the lgR (13), **(B)** The calculation of the lg(1/R) (14).

Supplementary Figure 7 Program for calculation of the ratio index. **(A)** The calculation of the 0-61 part of the ratio index (15), **(B)** The calculation of the 62–121 part of the ratio index (16), **(C)** The calculation of the 122–187 part of the ratio index (17). The programs for calculation of the normalized index are similar, except the R_*i*_/R_*j*_ was changed into (R_*i*_–R_*j*_)/(R_*i*_+R_*j*_).

Supplementary Figure 8 Program for calculation of the partial published index. **(A)** Published index 1–3 (18), **(B)** Published index 4–9 (19), **(C)** Published index 10–15 (20), **(D)** Published index 16–22 (21).

Supplementary Figure 9 Program for calculation of the partial published index. **(A)** Published index 23–31 (22), **(B)** Published index 32–35 (23), **(C)** Published index 36–43 (24), **(D)** Published index 44–56 (25).

Supplementary Figure 10 Program for calculation of the partial published index. **(A)** Published index 57–67 (26), **(B)** Published index 68–79 (27), **(C)** Published index 80–85 (28), **(D)** Published index 86–95 (29).

Supplementary Figure 11 Program for calculation of the correlation coefficient. **(A)** The program for calculation of the correlation coefficients between all the pigments and all the hyperspectral indices (30), **(B)** The program for combination the correlation coefficients of the ratio and normalized indices (31), **(C)** The program for building image with the correlation coefficients (32), **(D)** The program for finding the max correlation coefficient (33).

Supplementary Figure 12 Correlation coefficients between chlorophyll a and ratio lg(R) **(A)**, normalization lg(R) **(B)**, ratio d(lg(R)) **(C)**, normalization d(lg(R)) **(D)**, ratio dd(lg(R)) **(E)**, and normalization dd(lg(R)), **(F)** at the tillering stage.

Supplementary Figure 13 Correlation coefficients between chlorophyll a and ratio lg(1/R) **(A)**, normalization lg(1/R) **(B)**, ratio d(lg(1/R)) **(C)**, normalization d(lg(1/R)) **(D)**, ratio dd(lg(1/R)) **(E)**, and normalization dd(lg(1/R)) **(F)** at the tillering stage.

Supplementary Figure 14 Distribution of relative error of the single-variable models for the chlorophyll a **(A)**, chlorophyll b **(B)**, total chlorophyll **(C)**, and carotenoid **(D)** at the tillering stage. Distribution of relative error of the single-variable models for the chlorophyll a **(E)**, chlorophyll b **(F)**, total chlorophyll **(G)**, and carotenoid **(H)** at the heading stage.

Supplementary Figure 15 Distribution of relative error of the one independent variable **(A)**, two independent variables **(B)**, three independent variables **(C)**, four independent variables **(D)** models using stepwise regression analysis for chlorophyll a at the tillering stage.

Supplementary Table 1 The latest related papers of chlorophyll or nitrogen quantification with spectral methods.

Supplementary Table 2 Information about the 90 rice accessions and SPAD value.

Supplementary Table 3 Distribution of the pigments at the two stages.

Supplementary Table 4 Correlation coefficient (r) between the pigments at the two stages.

Supplementary Table 5 Spectral index based on spectral position and area.

Supplementary Table 6 Published spectral indices∗.

Supplementary Table 7 Comparison of performance between models using all of the indices and models using original indices.

Supplementary Table 8 Details of the multiple-variable models for 4 pigments.
