# Supplementary figures and images for: Accurate Digitization of the Chlorophyll Distribution of Individual Rice Leaves Using Hyperspectral Imaging and an Integrated Image Analysis Pipeline
Source: Front Plant Sci. 2017 Jul 25;8:1238. doi: 10.3389/fpls.2017.01238 (PMC5524744; doi:10.3389/fpls.2017.01238)

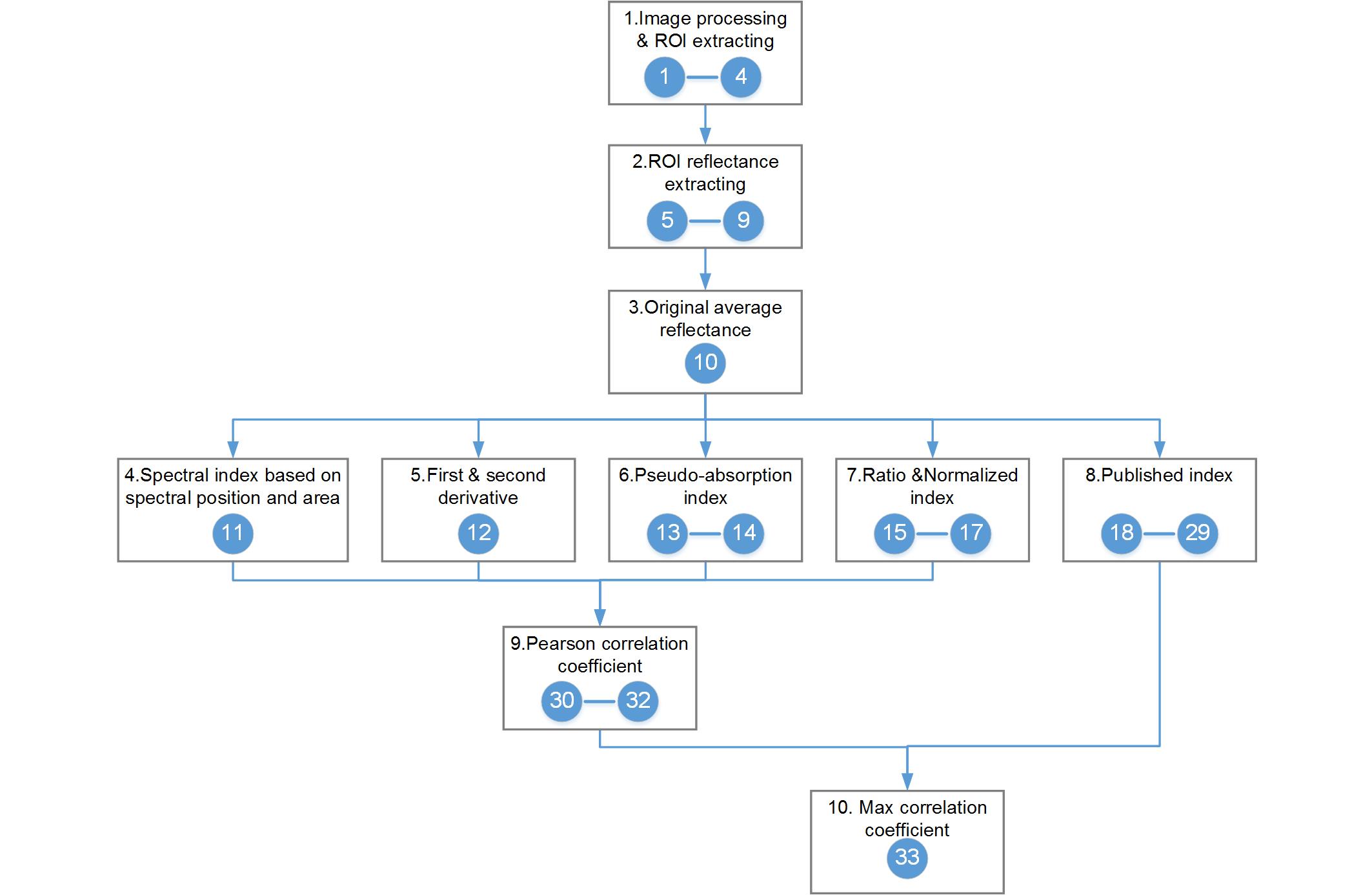

Supplement: Supplementary Figure 1 — Flow chart of the program. The number represents the processing module of the following Supplementary Figures 2–11. [file Image1.JPEG]

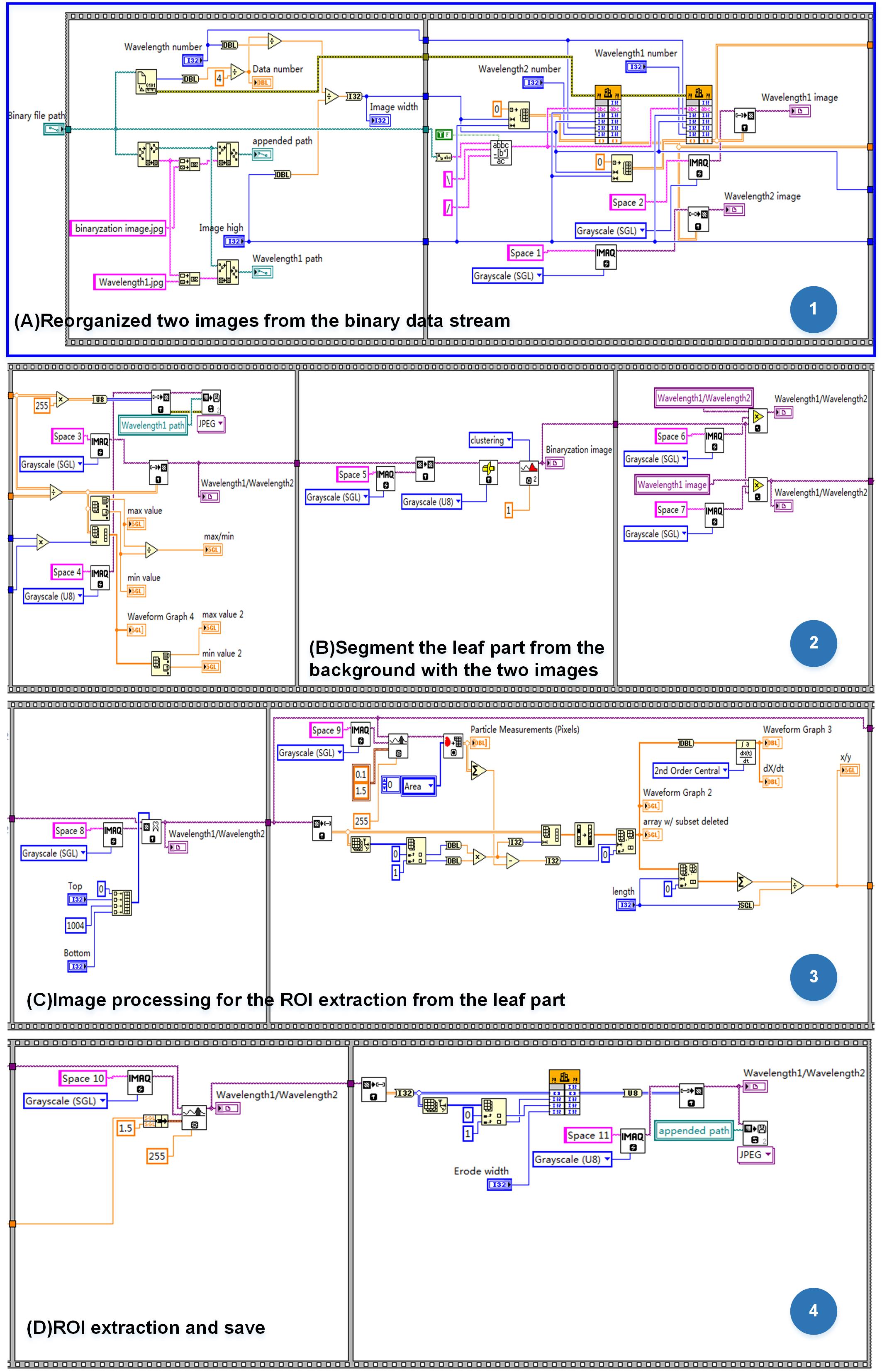

Supplement: Supplementary Figure 2 — Program for image processing and ROI extraction. (A) Reorganized two images from the binary data stream (1), (B) Segment the leaf part from the background with the two images (2), (C) Image processing for the ROI extraction from the leaf part (3), (D) ROI extraction and save (4). [file Image2.JPEG]

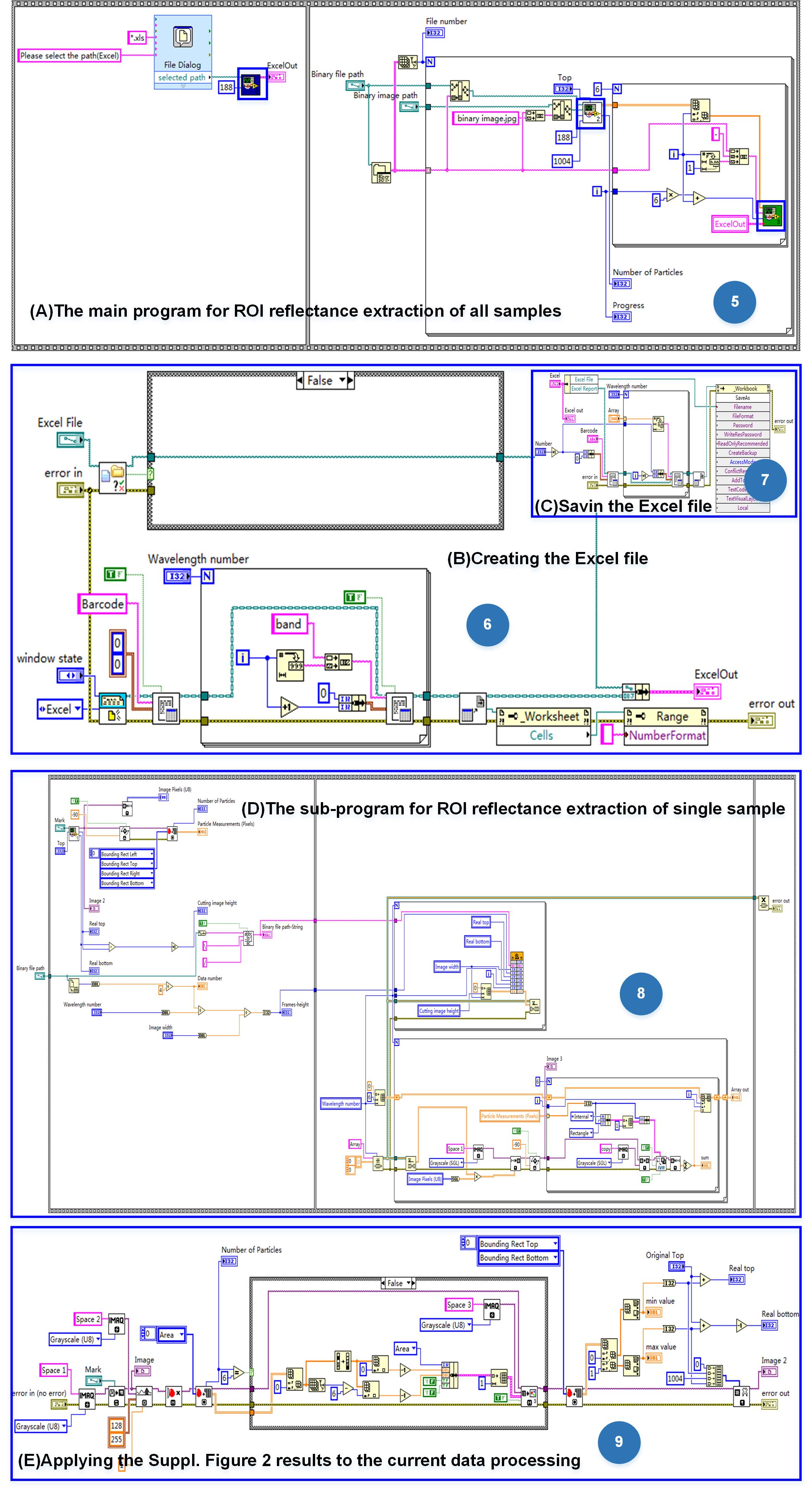

Supplement: Supplementary Figure 3 — Program for ROI reflectance extraction. (A) The main program for ROI reflectance extraction of all samples (5), (B) Creating the Excel file (6), (C) Savin the Excel file (7), (D) The sub-program for ROI reflectance extraction of single sample (8), (E) Applying the Supplementary Figure 2 results to the current data processing (9). [file Image3.JPEG]

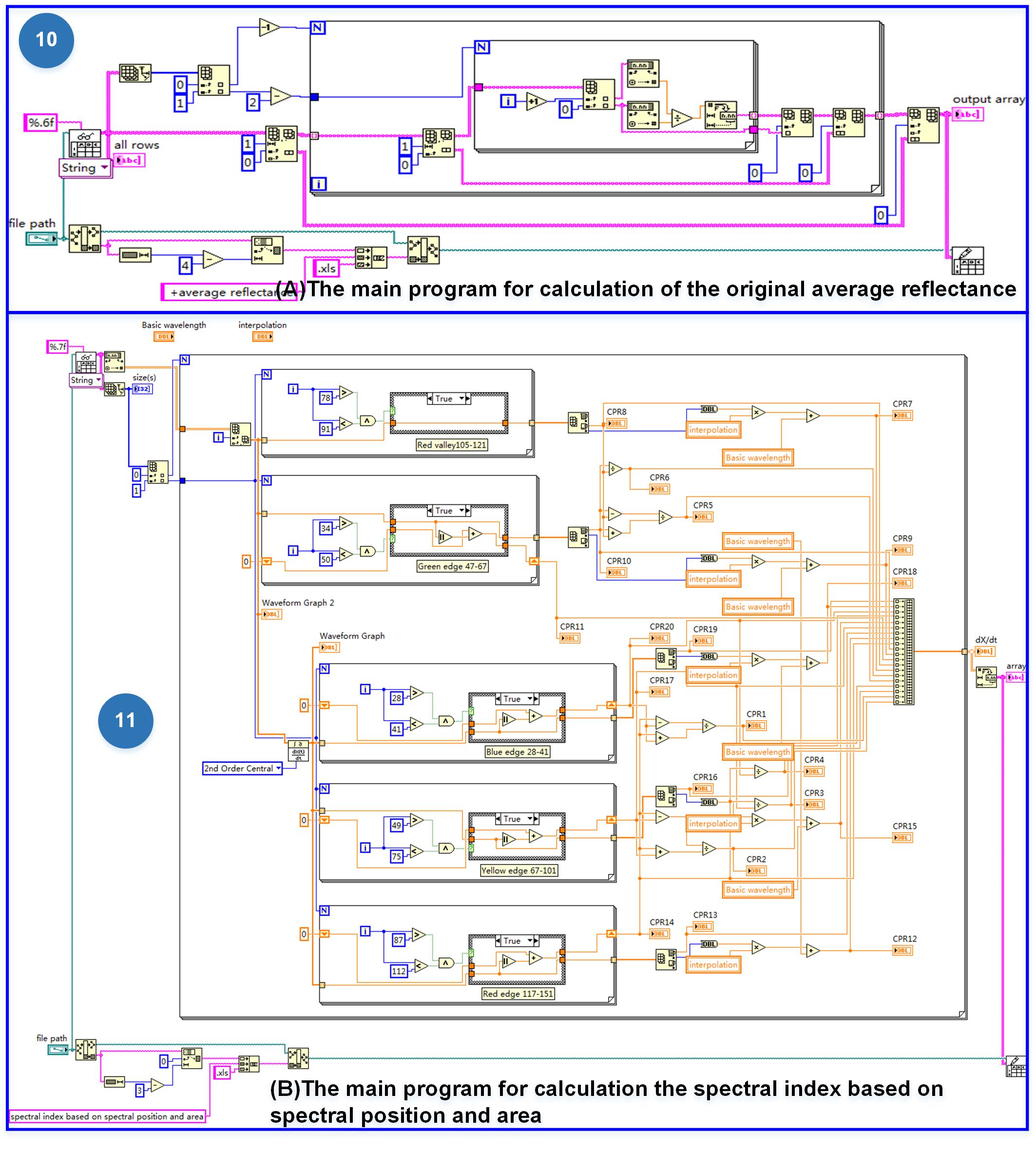

Supplement: Supplementary Figure 4 — Program for calculation of the original average reflectance (A) (10) and the spectral index based on spectral position and area (B) (11). [file Image4.JPEG]

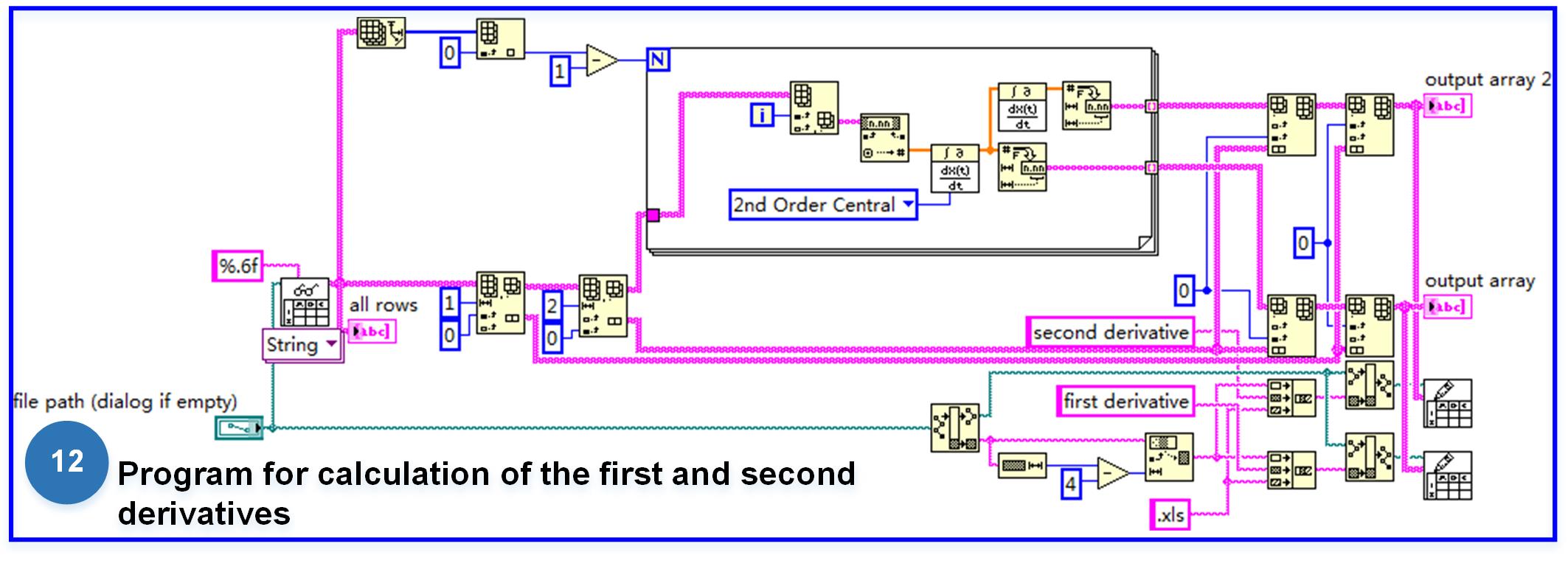

Supplement: Supplementary Figure 5 — Program for calculation of the first and second derivatives (12). [file Image5.JPEG]

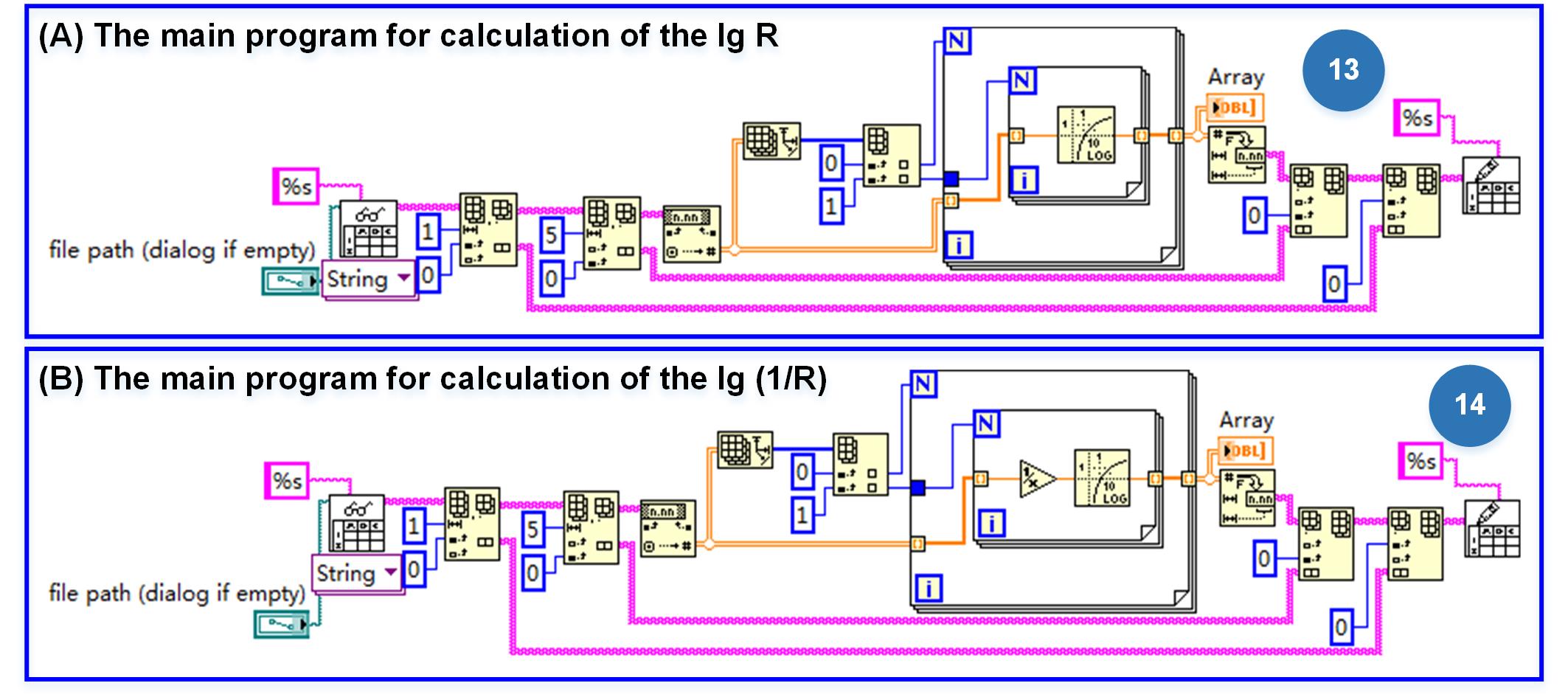

Supplement: Supplementary Figure 6 — Program for calculation of the pseudo-absorption index. (A) The calculation of the lgR (13), (B) The calculation of the lg(1/R) (14). [file Image6.JPEG]

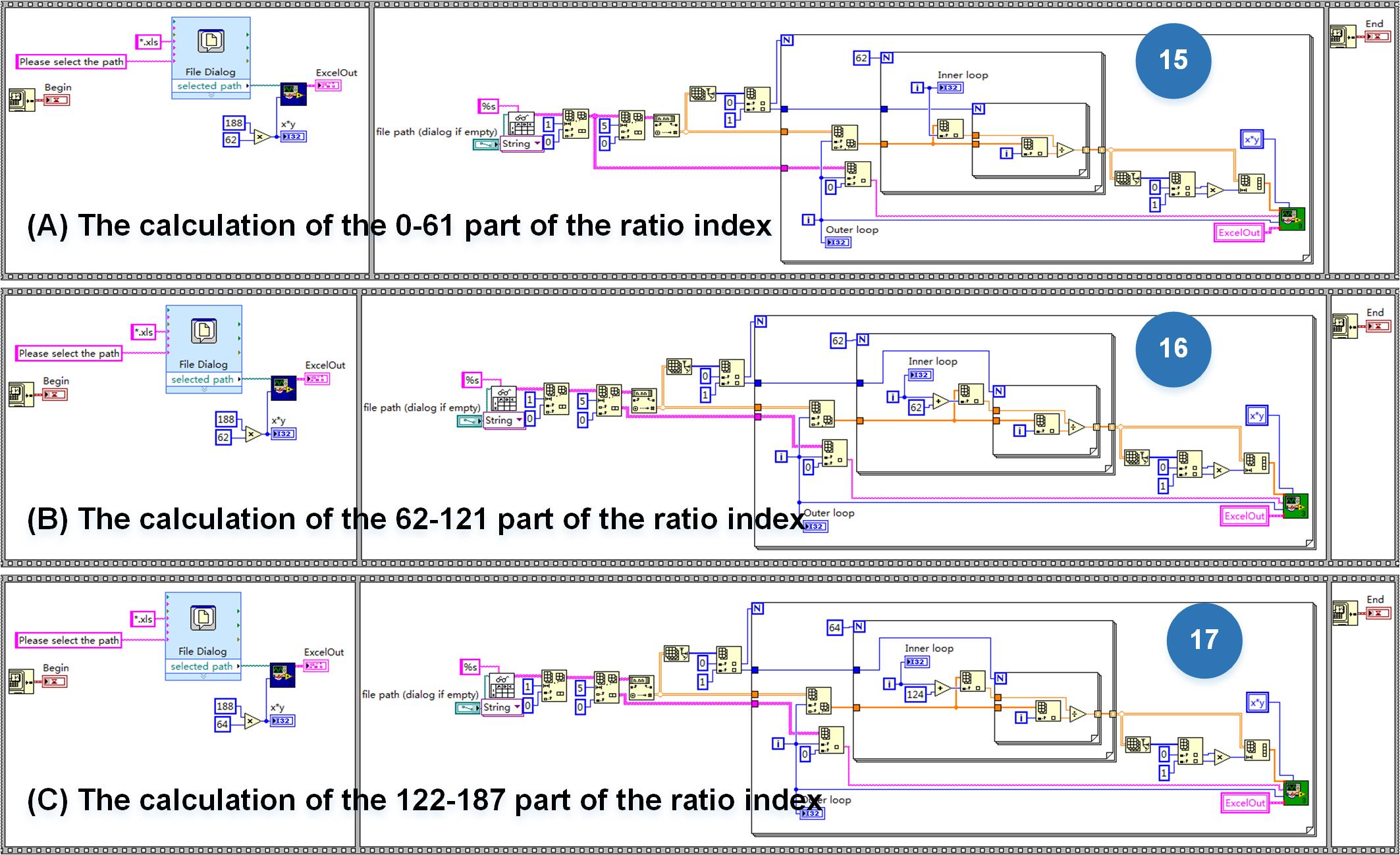

Supplement: Supplementary Figure 7 — Program for calculation of the ratio index. (A) The calculation of the 0-61 part of the ratio index (15), (B) The calculation of the 62–121 part of the ratio index (16), (C) The calculation of the 122–187 part of the ratio index (17). The programs for calculation of the normalized index are similar, except the Ri/Rj was changed into (Ri–Rj)/(Ri+Rj). [file Image7.JPEG]

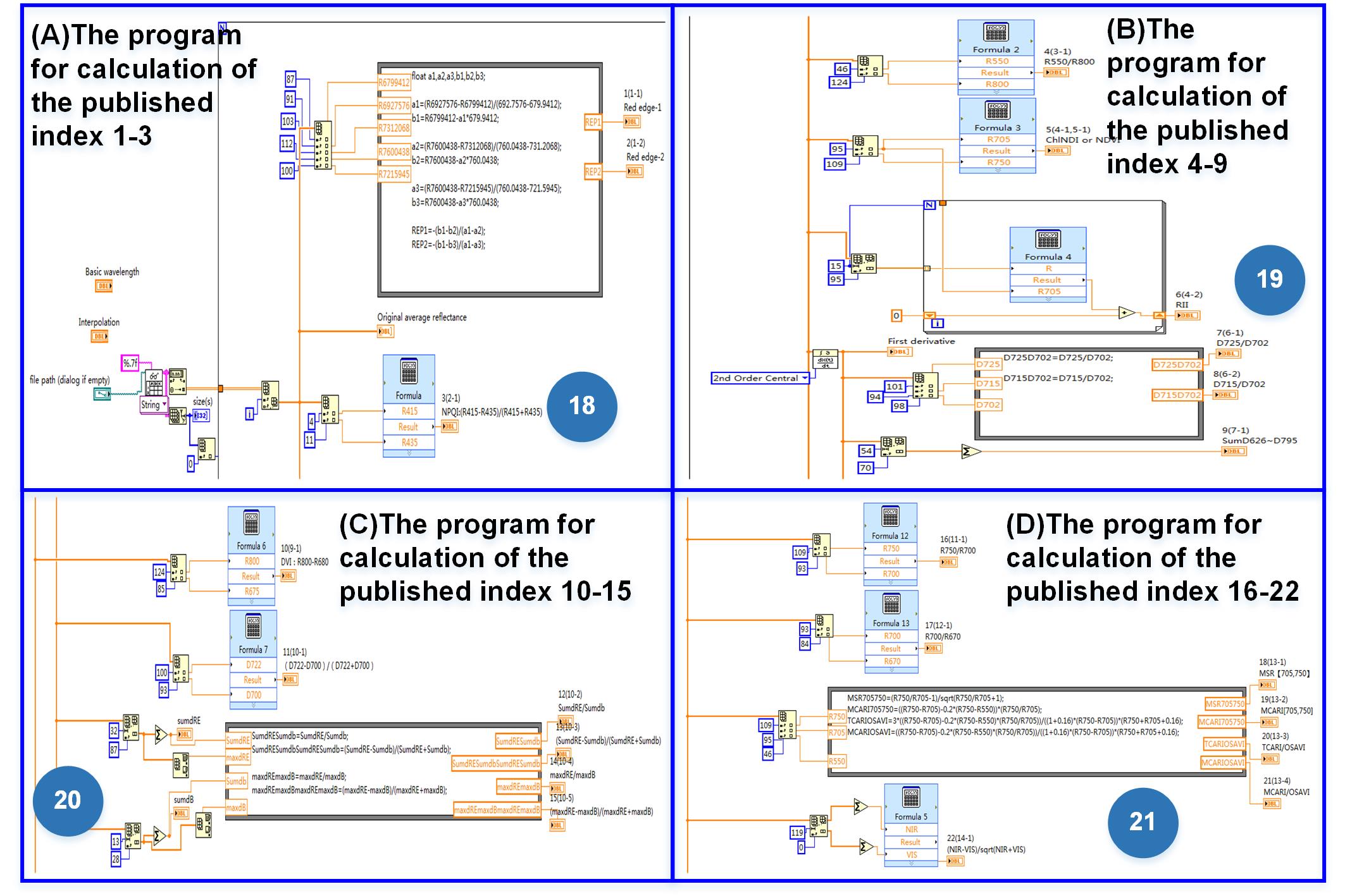

Supplement: Supplementary Figure 8 — Program for calculation of the partial published index. (A) Published index 1–3 (18), (B) Published index 4–9 (19), (C) Published index 10–15 (20), (D) Published index 16–22 (21). [file Image8.JPEG]

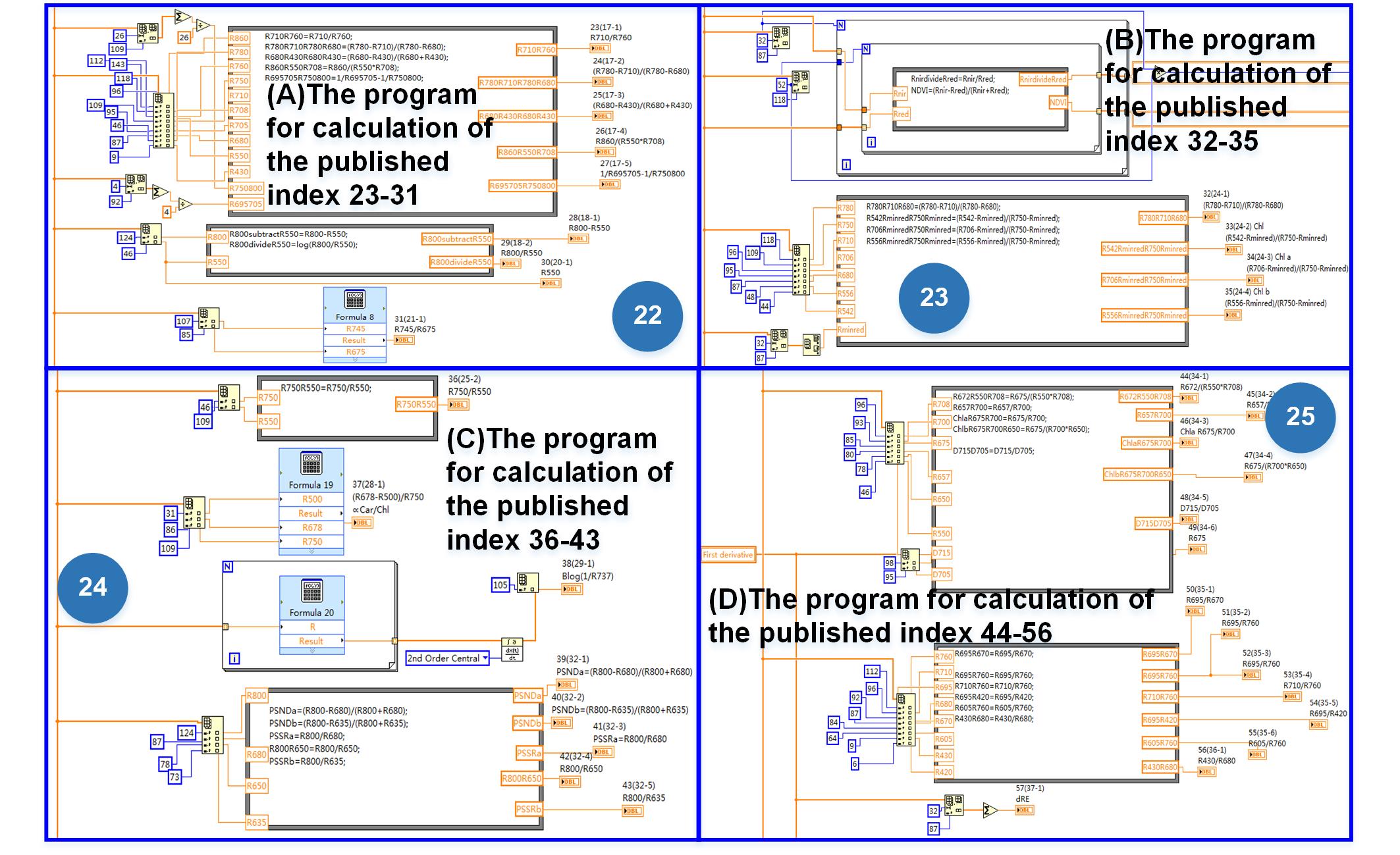

Supplement: Supplementary Figure 9 — Program for calculation of the partial published index. (A) Published index 23–31 (22), (B) Published index 32–35 (23), (C) Published index 36–43 (24), (D) Published index 44–56 (25). [file Image9.JPEG]

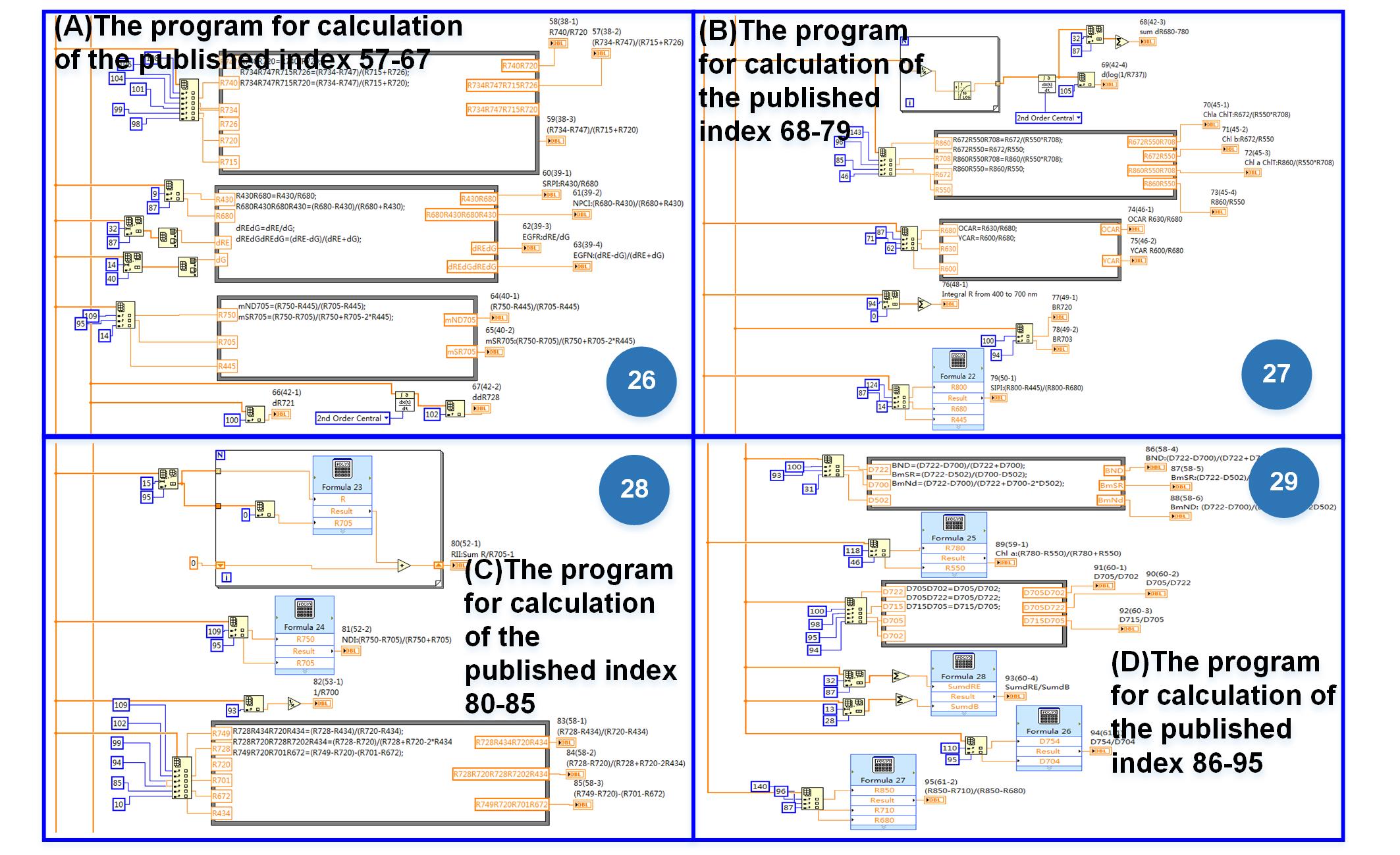

Supplement: Supplementary Figure 10 — Program for calculation of the partial published index. (A) Published index 57–67 (26), (B) Published index 68–79 (27), (C) Published index 80–85 (28), (D) Published index 86–95 (29). [file Image10.JPEG]

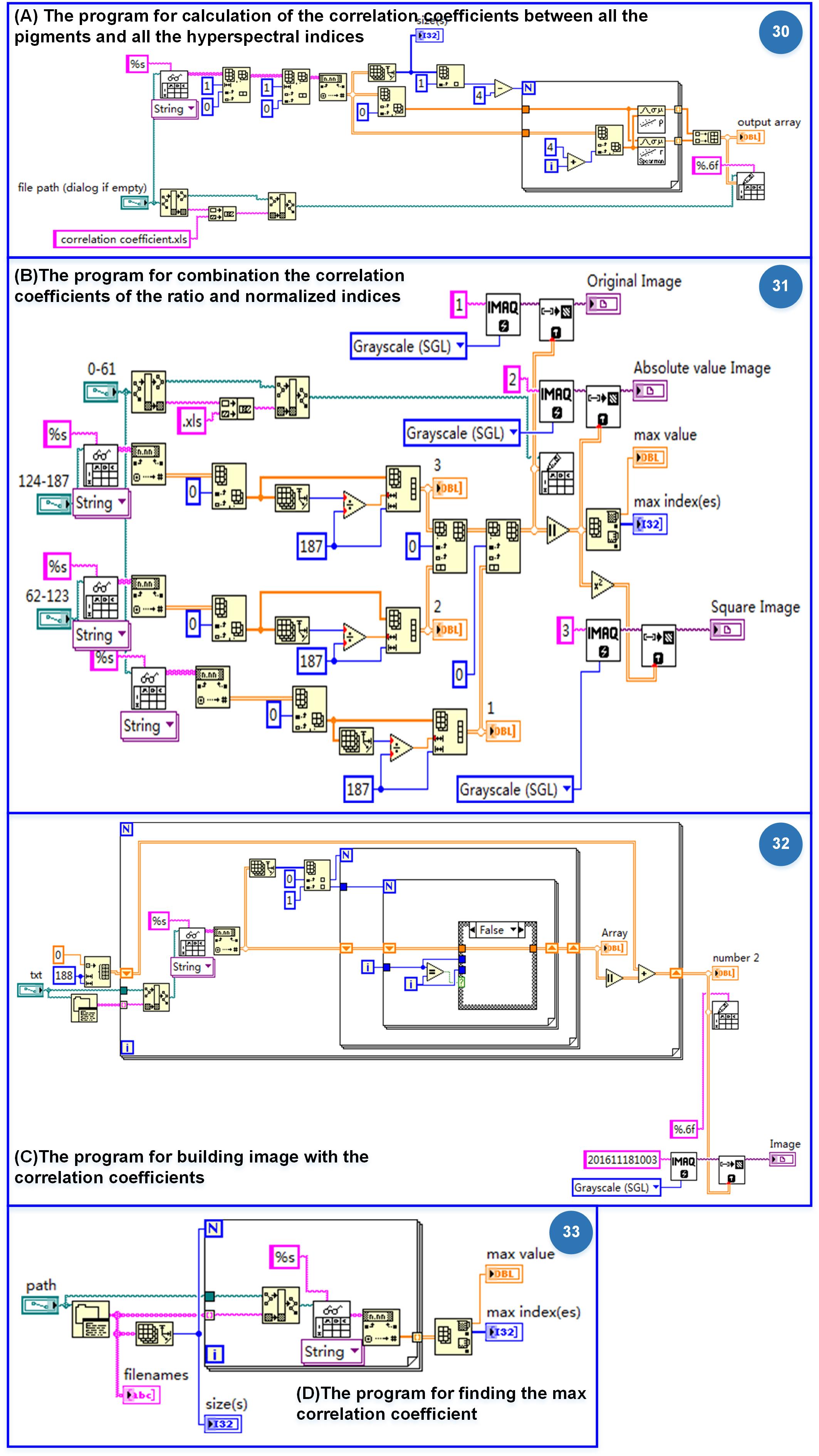

Supplement: Supplementary Figure 11 — Program for calculation of the correlation coefficient. (A) The program for calculation of the correlation coefficients between all the pigments and all the hyperspectral indices (30), (B) The program for combination the correlation coefficients of the ratio and normalized indices (31), (C) The program for building image with the correlation coefficients (32), (D) The program for finding the max correlation coefficient (33). [file Image11.JPEG]

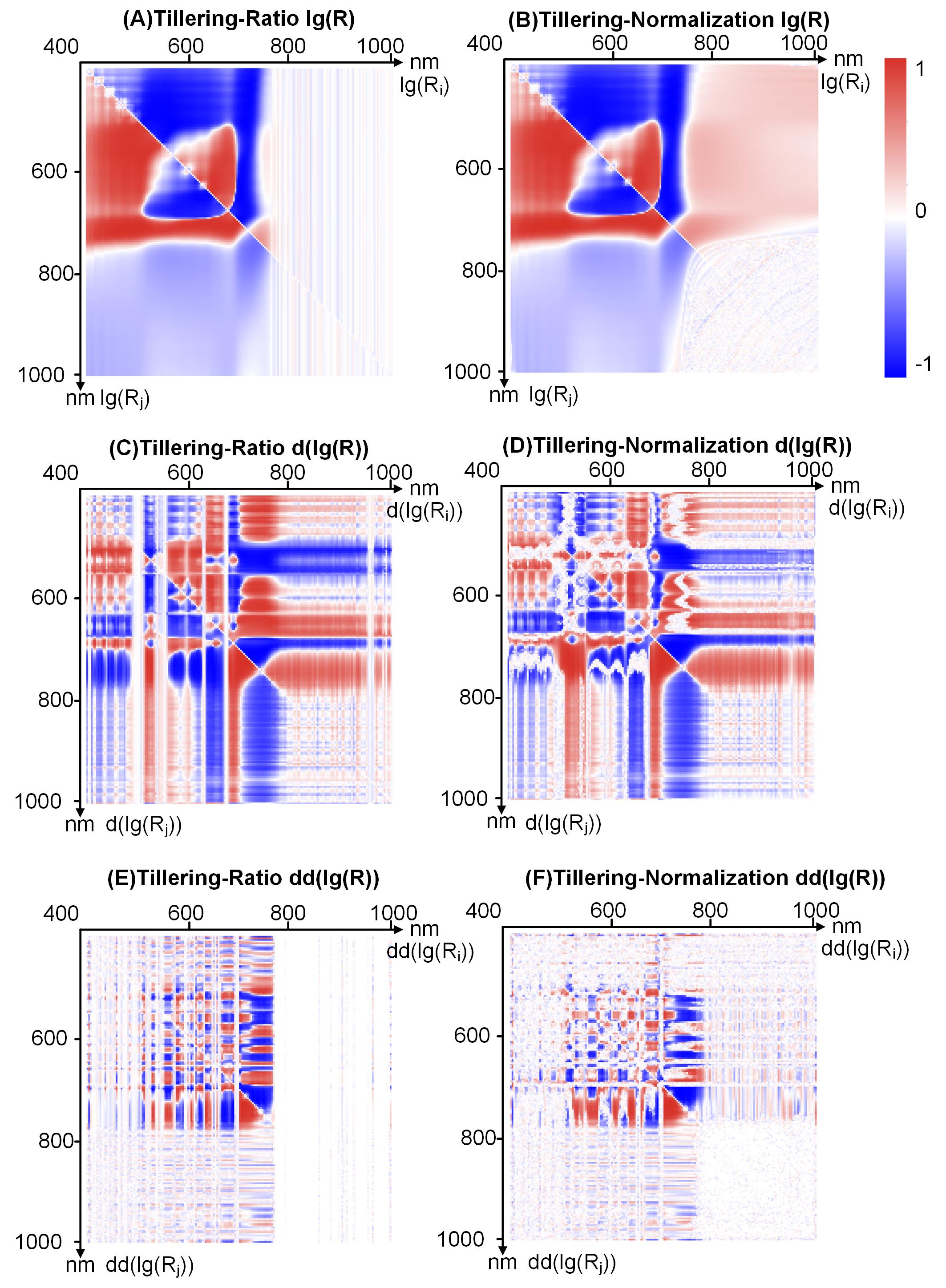

Supplement: Supplementary Figure 12 — Correlation coefficients between chlorophyll a and ratio lg(R) (A), normalization lg(R) (B), ratio d(lg(R)) (C), normalization d(lg(R)) (D), ratio dd(lg(R)) (E), and normalization dd(lg(R)), (F) at the tillering stage. [file Image12.JPEG]

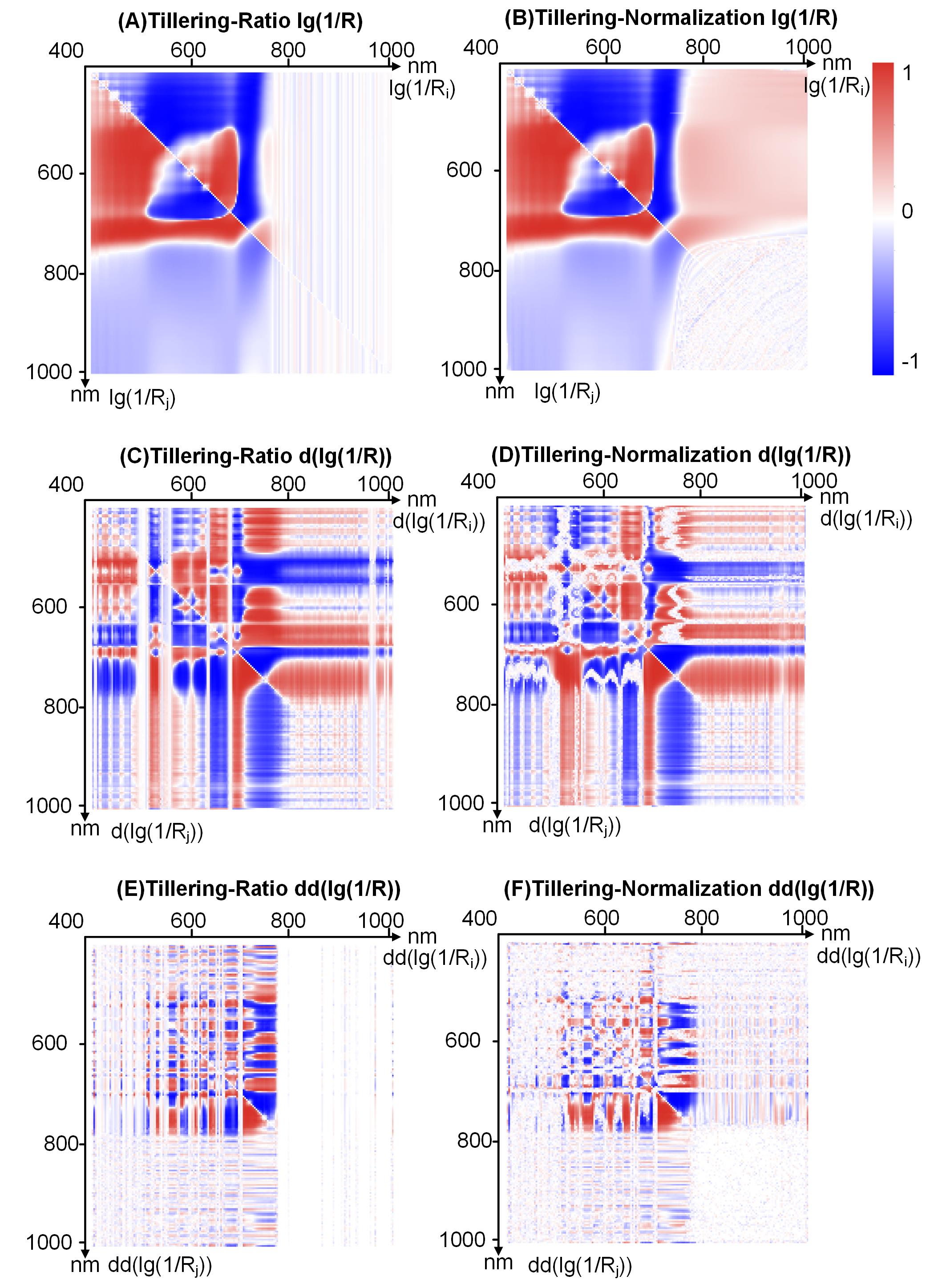

Supplement: Supplementary Figure 13 — Correlation coefficients between chlorophyll a and ratio lg(1/R) (A), normalization lg(1/R) (B), ratio d(lg(1/R)) (C), normalization d(lg(1/R)) (D), ratio dd(lg(1/R)) (E), and normalization dd(lg(1/R)) (F) at the tillering stage. [file Image13.JPEG]

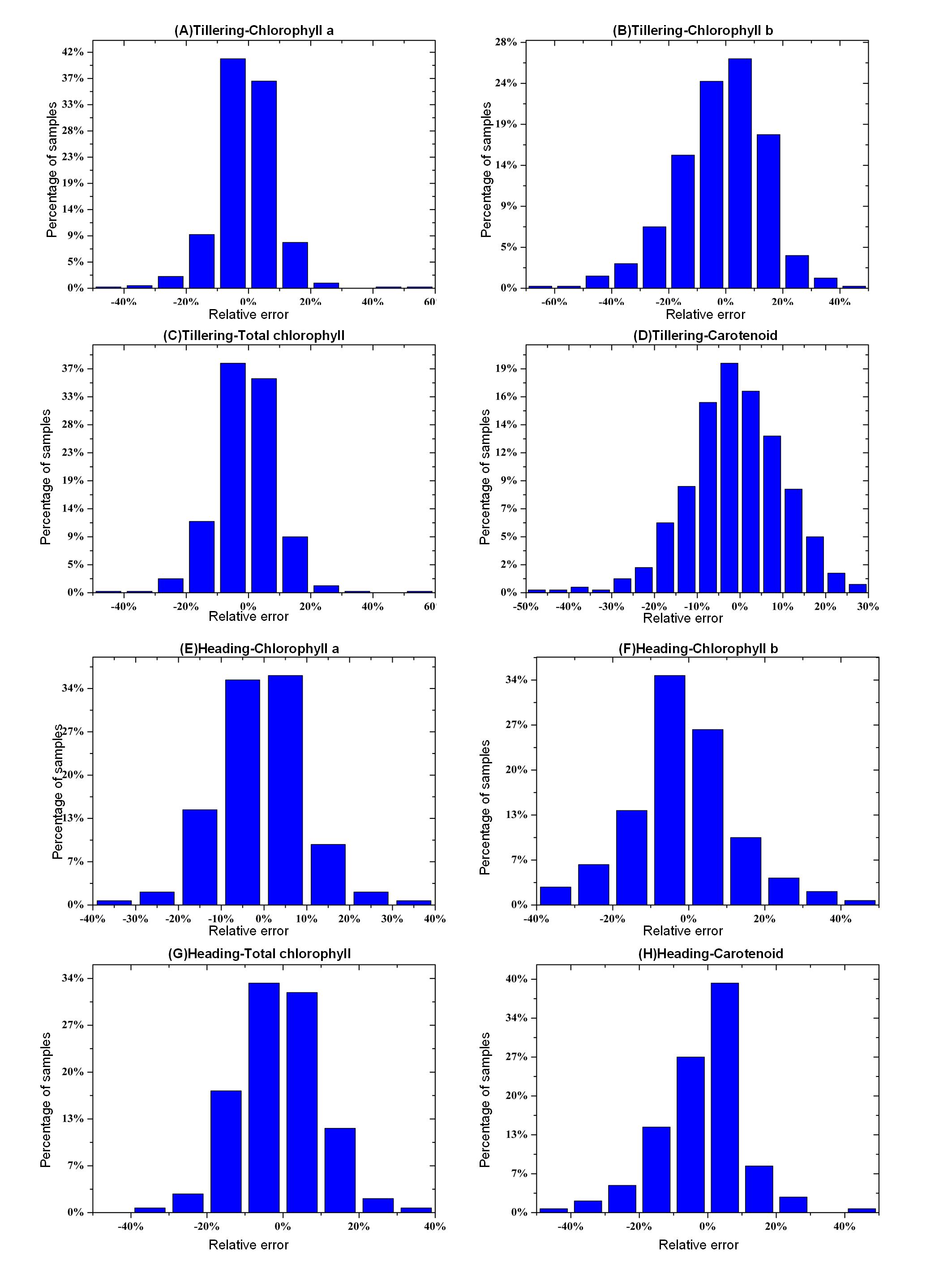

Supplement: Supplementary Figure 14 — Distribution of relative error of the single-variable models for the chlorophyll a (A), chlorophyll b (B), total chlorophyll (C), and carotenoid (D) at the tillering stage. Distribution of relative error of the single-variable models for the chlorophyll a (E), chlorophyll b (F), total chlorophyll (G), and carotenoid (H) at the heading stage. [file Image14.JPEG]

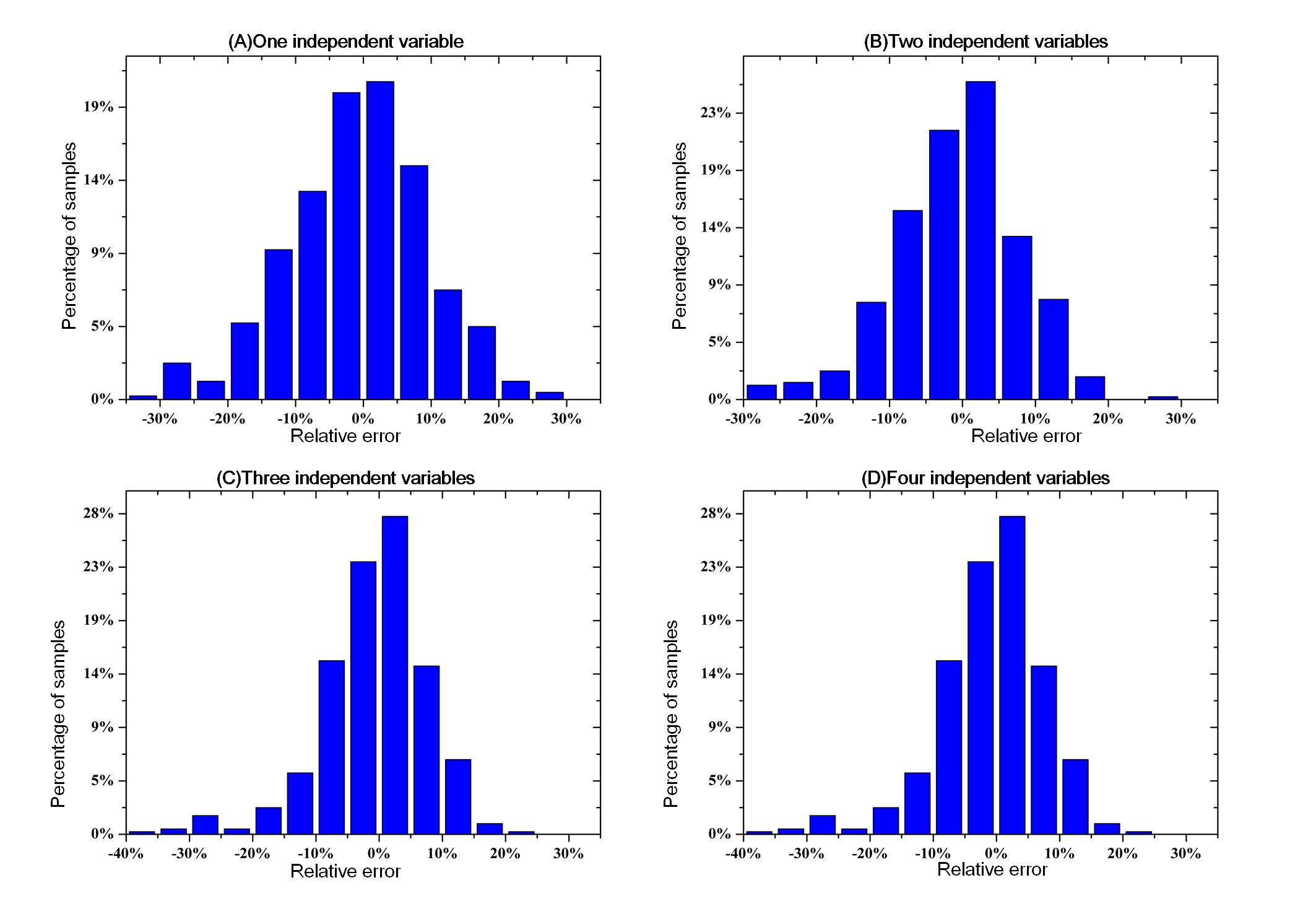

Supplement: Supplementary Figure 15 — Distribution of relative error of the one independent variable (A), two independent variables (B), three independent variables (C), four independent variables (D) models using stepwise regression analysis for chlorophyll a at the tillering stage. [file Image15.JPEG]
