# Supplementary material for: Accurate Digitization of the Chlorophyll Distribution of Individual Rice Leaves Using Hyperspectral Imaging and an Integrated Image Analysis Pipeline
Source: Front Plant Sci. 2017 Jul 25;8:1238. doi: 10.3389/fpls.2017.01238 (PMC5524744; doi:10.3389/fpls.2017.01238)
Supplement: Supplementary Table 1 — The latest related papers of chlorophyll or nitrogen quantification with spectral methods. [file Table1.DOCX]

**Supplementary Table** 1 The latest related papers of chlorophyll or nitrogen quantification with spectral methods.

| Species | Number of Samples | Level | Detector and spectral range | Important wavelength | Pigment | Manual measurement | R² | Chlorophyll/ nitrogen distribution | Data processing tool | Reference |
| --- | --- | --- | --- | --- | --- | --- | --- | --- | --- | --- |
| Cyanobacteria in turbid productive water | 55 | Remote sensing | 348-1074 nm  ASD FieldSpec UV–VNIR spectroradiometer | 620 nm, 665 nm | Chlorophyll a  phycocyanin | TD-700 Fluorometer | 0.40-0.77 | -- | -- | ([Randolph et al., 2008](#_ENREF_20)) |
| Grass and herb species | 45 | Canopy | 350-2500 nm  GER 3700 spectroradiometer | --  (red edge inflection point (REIP), stepwise regression and partial least squares regression) | Total chlorophyll | SPAD-502 Leaf Chlorophyll Meter | 0.25-0.69 | -- | MATLAB | ([Darvishzadeh et al., 2008](#_ENREF_3)) |
| 9 crops (garlic, alfalfa, onion, sunflower, corn, potato, sugar beet, vineyard and wheat) | 131 | Remote sensing | 400-1050 nm  CHRIS (Compact High Resolution Imaging Spectrometer) | 643-795 nm | Total chlorophyll | CCM-200 Chlorophyll Content Meter | 0.83 | Yes | -- | ([Delegido et al., 2010](#_ENREF_4)) |
| Cucumber | 100 | Leaf | 400-1000 nm  hyperspectral imaging system （Specim V10E） | 680-780 nm | Total chlorophyll | Spectrophotometer | 0.69 | Yes | MATLAB  ENVI | ([Zou et al., 2011](#_ENREF_34)) |
| Cucumber | 120 | Leaf | 900-1700 nm  hyperspectral imaging system （ImSpector, VI7E, Spectra Imaging） | --  (PCA) | Chlorophyll a  Chlorophyll b  Total chlorophyll | HPLC | 0.76 | Yes | MATLAB  ENVI | ([Shi et al., 2012](#_ENREF_21)) |
| Urban vegetation: London plane, Canary Island date palm, European nettle tree and White mul-berry | 320 | Canopy | 350-1200 nm  CASI imagery | 643-795 nm | Chlorophyll a  Chlorophyll b  Total chlorophyll | Spectrophotometer, SPAD-502 chlorophyll meter | 0.87-0.92 | Yes | -- | ([Delegido et al., 2014](#_ENREF_5)) |
| Scots pine shoots |  | Leaf | 400-2200 nm  hyperspectral LiDAR instrument | 705 nm, 750 nm | Chlorophyll a  Chlorophyll b  Total chlorophyll | Porra method | 0.88 | Yes | -- | ([Nevalainen et al., 2014](#_ENREF_18)) |
| Chlorophyll a in lake | 38 | Canopy | 350-1075 nm  ASD FieldSpec Spectrophotometer | 653 nm, 691 nm, and 748 nm | Chlorophyll a | UV-2501 spectrophotometer | 0.57-0.85 | Yes | MATLAB | ([Zhou et al., 2014](#_ENREF_32)) |
| Cotton | 193 | Canopy | 350-2500 nm  ASD FieldSpec FR | 515-550 nm, 715 nm, and 750 nm | Carotenoids | Spectrophotometer | 0.456-0.563 | -- | -- | ([Yi et al., 2014](#_ENREF_29)) |
| Winter wheat | 218 | Canopy | 300-1150 nm  Passive spectrometer (tec5,Oberursel,Germany) | 662 nm, 682 nm | Nitrogen | -- | 0.79-0.90 | -- | MATLAB | ([Li et al., 2014](#_ENREF_14)) |
| Barley and synthetic | 294 | Canopy | 350-1800 nm  QualitySpec Pro and FieldSpec 3 spectroradiometers | 505 nm, 515nm, 525 nm, 535 nm,540 nm, 555 nm, 570 nm, 615 nm, 675 nm, 700 nm, 725 nm, 730 nm, 735 nm, 785 nm | Total chlorophyll | UV–VIS spectrophotometer | 0.65-0.98 | -- | SAS | ([Yu et al., 2014](#_ENREF_30)) |
| Mosses and vascular plants | 26 | Leaf | 325-1075 nm  Compact Airborne Spectrographic Imager 2 (CASI-2) | 705 nm and red edge | Total chlorophyll  Nitrogen | Elemental analyzer | 0.29-0.32 | Yes | -- | ([Kalacska et al., 2015](#_ENREF_12)) |
| Black spruce and trembling aspen | 600 | Leaf  Canopy | 400-2500 nm  ASD spectroradiometer Fieldspec Pro FR | --  PROSPECT model: Blue 456-475 nm, Green 544-564 nm, Red 620-670 nm, NIR 837-876 nm, SWIR 1616-1644 nm | Total chlorophyll | Cary-1 Spectrophotometer | 0.75-0.99 | Yes | GeoDa software package | ([Croft et al., 2015](#_ENREF_2)) |
| Potato | 270 | Leaf | 401-982 nm  Airborne Imaging Spectrometer for Applications (AISA Eagle) | 713 nm, 751 nm | Nitrogen | Combustion analyzer | 0.77-0.79 | -- | ENVI | ([Nigon et al., 2015](#_ENREF_19)) |
| Rice | 150 | Canopy | 350-1000 nm  ASD Field Spec Pro spectrometer | 640 nm, 732 nm and 752 nm | Total chlorophyll | SPAD-502 | 0.89 | Yes | -- | ([Li et al., 2015](#_ENREF_16)) |
| Maple, Chestnut, Beech | 90 | Leaf | 400-800 nm  Hitachi 150–20 spectrophotometer, 2101 PC spectrophotometer | --  (NN and PLS) | Total chlorophyll  Carotenoids | Reflectance measurement | 0.70-0.97 | -- | -- | ([Kira et al., 2015](#_ENREF_13)) |
| Lettuce | 140 | Leaf | 380-1012 nm  A-Series VNIR Micro-Hyperspec Sensor  (Headwall Photonics, Fitchburg, MA, USA) | 677 nm, 744 nm, 904 nm | Total chlorophyll | PCSTestr 35 | 0.92 | -- | -- | ([Simko et al., 2015](#_ENREF_22)) |
| Winter wheat | 407 | Remote sensing | 325-1075 nm  ASD FieldSpec Handheld spectrometer (Analytical Spectral Devices Inc., USA) | 419 nm, 742 nm and 759 nm | Total chlorophyll  Nitrogen | Micro-Kjeldahl method | 0.788-0.813 | -- | MATLAB | ([Feng et al., 2015](#_ENREF_7)) |
| Ponds (surface water) | 120 | Canopy | 400-1000 nm  An imaging spectrograph (Imspector V10E, SpectralImagingLtd., Oulu,Finland) | 666 nm, 702 nm | Chlorophyll a | Spectrophotometer | 0.82-0.96 | Yes | -- | ([Wang et al., 2016](#_ENREF_26)) |
| Winter oilseed rape |  | Canopy | 350-2500 nm  Analytical Spectral Devices Field Spec Pro spectrometer (ASD, Boulder, CO, USA) | 680-760 nm | Total chlorophyll | UV-5200 spectrophotometer | 0.88 | -- | -- | ([Li et al., 2016](#_ENREF_15)) |
| Rice | 24 | Canopy  Remote sensing | 350-1050 nm  400-2500 nm  portable spectroradiometers (FieldSpec-FR, ASD)  EO-1 Satellite of Hyperion sensor | 491 nm, 574 nm, 681 nm, 695 nm, 705 nm, 709 nm, 717 nm, 740 nm, 750 nm, 800 nm | Chlorophyll a  Chlorophyll b  Total chlorophyll  Nitrogen | Spectrophotometer, titration | 0.69-0.82 | Yes | ENVI | ([Moharana and Dutta, 2016](#_ENREF_17)) |
| Rice | 30 | Leaf | 300-920 nm  a photosensitive APD array | --  (SVM) | Nitrogen | Kjeldahl analysis | 0.51-0.75 | -- | -- | ([Du et al., 2016](#_ENREF_6)) |
| Winter wheat | 237 | Canopy | 325-1075 nm  ASD FieldSpec HandHeld data-logger (Analytical Spectral Devices, Boulder, CO, USA) | Red-edge VIs (e.g., mND705, GND [750,550], NDRE, RI-1dB) | Nitrogen | Kjeldahl apparatus | 0.74-0.87 | -- | MATLAB | ([He et al., 2016a](#_ENREF_10)) |
| Winter wheat | 174  63 | Canopy | 325-1075 nm  ASD FieldSpec HandHeld(Analytical Spectral Devices, Boulder, CO, USA) | 478 nm, 538 nm, 634 nm, 768 nm | Nitrogen | Micro-Kjeldahl method | 0.819-0.897 | -- | MATLAB | ([He et al., 2016b](#_ENREF_11)) |
| Winter wheat | 602 | Canopy | 325-1075 nm  ASD FieldSpec Handheld spec-trometer (Analytical Spectral Devices Inc., USA) | 680 nm, 700 nm, 725 nm, 756 nm | Nitrogen | Micro-Kjeldahl method | 0.831 | -- | MATLAB | ([Guo et al., 2016](#_ENREF_9)) |
| Winter wheat | 402 | Remote sensing | 325-1075 nm  ASD Field Spec Pro spectrometer (Analytical Spectral Devices, USA) | WRNI: [(R_735_ −R_720_)*R_900_]/[R_min_(R_930_ −R_980_)*(R_735_ + R_720_)] | Nitrogen | Dumas Elementary Analyser | 0.818-0.843 | -- | MATLAB | ([Feng et al., 2016](#_ENREF_8)) |
| Water (Microcystis algae blooms) | 29 | Remote sensing | 371-1042 nm  A CASI-1500 VNIR airborne hyperspectral imaging system | 686 nm, 714 nm | Chlorophyll a | 10200H-2.b: Spectrophotometric | 0.12-0.84 | Yes | ENVI | ([Beck et al., 2016](#_ENREF_1)) |
| Corn | 66 | Leaf | 400-1000 nm  Spectrometer | --  (PLS) | Total chlorophyll | SPAD-502 meter | 0.612 | -- | MATLAB  ENVI | ([Wu et al., 2016](#_ENREF_28)) |
| sunlit leaves, shaded leaves | 551 | Leaf | 400-2500 nm  FieldSpec spectrometer | 550 nm, 672 nm, 708 nm | Total chlorophyll | UV-VIS spectrophotometer | 0.23-0.71 | -- | -- | ([Sonobe and Wang, 2016](#_ENREF_23)) |
| Winter wheat | 59 | Remote sensing | 350-2500 nm  ASD FieldSpec Pro spectrometer (Analytical Spectral Devices, Boulder, CO, USA) | 705 nm and red edge | Nitrogen | Kjeldahl method | 0.77-0.96 | -- | -- | ([Zhou et al., 2016](#_ENREF_33)) |
| Durum wheat | 2103 | Canopy | 268-1095 nm  portable field spectroradiometer (GER 1500, Spectra Vista Corp., Poughkeepsie, New York) | 583-722 nm | Nitrogen | Carlo Erba elemental analyzer | 19.3%≤RMSECV≤ 36.3%. | -- | R | ([Thorp et al., 2017](#_ENREF_24)) |
| Spinach | 180 | Leaf | 874-1734 nm  imaging spectrograph (ImSpector N17E; Spectral Imaging Ltd., Oulu, Finland) | --  (PLS) | Chlorophyll a  Chlorophyll b  Total chlorophyll  Carotenoids | Spectrophotometer | 0.66  0.66  0.71  0.79 | Yes | -- | ([Zhang et al., 2017](#_ENREF_31)) |
| Pure broadleaf, pure needle leaf, and mixed forest | 26 | Remote sensing | 400-2500 nm  Two imaging spectrometers | --  (continuous wavelet analysis (CWA), PLSR) | Nitrogen | Measured in the laboratory | 0.65 | -- | MATLAB | ([Wang et al., 2017](#_ENREF_27)) |
| Grasses, forbs, and shrubs | 128 | leaf and canopy | 405-950 nm  Analytical Spectral Devices (ASD) FieldSpec 3 Max field portable spectroradiometer (Analytical Spectral Devices, Inc., Boulder, Colorado, USA) and an ASD Pro Lamp (Analytical Spectral Devices, Inc., Boulder, Colorado, USA) | 690-750 nm | Chlorophyll a  Chlorophyll b  Total chlorophyll | Spectrophotometer | 0.24-0.67 | Yes | -- | ([Tong and He, 2017](#_ENREF_25)) |
| Rice | 300 | Leaf | 400-1000 nm  hyperspectral imaging spectrometers (HyperspecTM VNIR, Headwall Photonics, USA) | 700-760 nm | Chlorophyll a  Chlorophyll b  Total chlorophyll  Carotenoid | Spectrophotometer | 0.654-0.928 | Yes | Image pipeline with LabVIEW | This present work in this article |

**References**

Beck, R., Zhan, S., Liu, H., Tong, S., Yang, B., Xu, M., Ye, Z., Huang, Y., Shu, S., Wu, Q., Wang, S., Berling, K., Murray, A., Emery, E., Reif, M., Harwood, J., Young, J., Nietch, C., Macke, D., Martin, M., Stillings, G., Stump, R., and Su, H. (2016). Comparison of satellite reflectance algorithms for estimating chlorophyll-a in a temperate reservoir using coincident hyperspectral aircraft imagery and dense coincident surface observations. *Remote Sensing of Environment* 178**,** 15-30.

Croft, H., Chen, J.M., Zhang, Y., Simic, A., Noland, T.L., Nesbitt, N., and Arabian, J. (2015). Evaluating leaf chlorophyll content prediction from multispectral remote sensing data within a physically-based modelling framework. *Isprs Journal of Photogrammetry and Remote Sensing* 102**,** 85-95.

Darvishzadeh, R., Skidmore, A., Schlerf, M., Atzberger, C., Corsi, F., and Cho, M. (2008). LAI and chlorophyll estimation for a heterogeneous grassland using hyperspectral measurements. *Isprs Journal of Photogrammetry and Remote Sensing* 63**,** 409-426.

Delegido, J., Alonso, L., Gonzalez, G., and Moreno, J. (2010). Estimating chlorophyll content of crops from hyperspectral data using a normalized area over reflectance curve (NAOC). *International Journal of Applied Earth Observation and Geoinformation* 12**,** 165-174.

Delegido, J., Van Wittenberghe, S., Verrelst, J., Ortiz, V., Veroustraete, F., Valcke, R., Samson, R., Rivera, J.P., Tenjo, C., and Moreno, J. (2014). Chlorophyll content mapping of urban vegetation in the city of Valencia based on the hyperspectral NAOC index. *Ecological Indicators* 40**,** 34-42.

Du, L., Gong, W., Shi, S., Yang, J., Sun, J., Zhu, B., and Song, S. (2016). Estimation of rice leaf nitrogen contents based on hyperspectral LIDAR. *International Journal of Applied Earth Observation and Geoinformation* 44**,** 136-143.

Feng, W., Guo, B., Zhang, H., He, L., Zhang, Y., Wang, Y., Zhu, Y., and Guo, T. (2015). Remote estimation of above ground nitrogen uptake during vegetative growth in winter wheat using hyperspectral red-edge ratio data. *Field Crops Research* 180**,** 197-206.

Feng, W., Zhang, H., Zhang, Y., Qi, S., Heng, Y., Guo, B., Ma, D., and Guo, T. (2016). Remote detection of canopy leaf nitrogen concentration in winter wheat by using water resistance vegetation indices from in-situ hyperspectral data. *Field Crops Research* 198**,** 238-246.

Guo, B., Qi, S., Heng, Y., Duan, J., Zhang, H., Wu, Y., Feng, W., Xie, Y., and Zhu, Y. (2016). Remotely assessing leaf N uptake in winter wheat based on canopy hyperspectral red-edge absorption. *European Journal of Agronomy* 82.

He, L., Song, X., Feng, W., Guo, B., Zhang, Y., Wang, Y., Wang, C., and Guo, T. (2016a). Improved remote sensing of leaf nitrogen concentration in winter wheat using multi-angular hyperspectral data. *Remote Sensing of Environment* 174**,** 122-133.

He, L., Zhang, H., Zhang, Y., Song, X., Feng, W., Kang, G., Wang, C., and Guo, T. (2016b). Estimating canopy leaf nitrogen concentration in winter wheat based on multi-angular hyperspectral remote sensing. *European Journal of Agronomy* 73**,** 170-185.

Kalacska, M., Lalonde, M., and Moore, T.R. (2015). Estimation of foliar chlorophyll and nitrogen content in an ombrotrophic bog from hyperspectral data: Scaling from leaf to image. *Remote Sensing of Environment* 169**,** 270-279.

Kira, O., Linker, R., and Gitelson, A. (2015). Non-destructive estimation of foliar chlorophyll and carotenoid contents: Focus on informative spectral bands. *International Journal of Applied Earth Observation and Geoinformation* 38**,** 251-260.

Li, F., Mistele, B., Hu, Y., Chen, X., and Schmidhalter, U. (2014). Reflectance estimation of canopy nitrogen content in winter wheat using optimised hyperspectral spectral indices and partial least squares regression. *European Journal of Agronomy* 52**,** 198-209.

Li, L., Ren, T., Ma, Y., Wei, Q., Wang, S., Li, X., Cong, R., Liu, S., and Lu, J. (2016). Evaluating chlorophyll density in winter oilseed rape (Brassica napus L.) using canopy hyperspectral red-edge parameters. *Computers and Electronics in Agriculture* 126**,** 21-31.

Li, X., Liu, X., Liu, M., Wang, C., and Xia, X. (2015). A hyperspectral index sensitive to subtle changes in the canopy chlorophyll content under arsenic stress. *International Journal of Applied Earth Observation & Geoinformation* 36**,** 41-53.

Moharana, S., and Dutta, S. (2016). Spatial variability of chlorophyll and nitrogen content of rice from hyperspectral imagery. *Isprs Journal of Photogrammetry and Remote Sensing* 122**,** 17-29.

Nevalainen, O., Hakala, T., Suomalainen, J., Makipaa, R., Peltoniemi, M., Krooks, A., and Kaasalainen, S. (2014). Fast and nondestructive method for leaf level chlorophyll estimation using hyperspectral LiDAR. *Agricultural and Forest Meteorology* 198**,** 250-258.

Nigon, T.J., Mulla, D.J., Rosen, C.J., Cohen, Y., Alchanatis, V., Knight, J., and Rud, R. (2015). Hyperspectral aerial imagery for detecting nitrogen stress in two potato cultivars. *Computers and Electronics in Agriculture* 112**,** 36-46.

Randolph, K., Wilson, J., Tedesco, L., Li, L., Pascual, D.L., and Soyeux, E. (2008). Hyperspectral remote sensing of cyanobacteria in turbid productive water using optically active pigments, chlorophyll a and phycocyanin. *Remote Sensing of Environment* 112**,** 4009-4019.

Shi, J., Zou, X., Zhao, J., Wang, K., Chen, Z., Huang, X., Zhang, D., and Holmes, M. (2012). Nondestructive diagnostics of nitrogen deficiency by cucumber leaf chlorophyll distribution map based on near infrared hyperspectral imaging. *Scientia Horticulturae* 138**,** 190-197.

Simko, I., Jimenezberni, J., and Furbank, R. (2015). Detection of decay in fresh-cut lettuce using hyperspectral imaging and chlorophyll fluorescence imaging. *Postharvest Biology and Technology* 106**,** 44-52.

Sonobe, R., and Wang, Q. (2016). Hyperspectral indices for quantifying leaf chlorophyll concentrations performed differently with different leaf types in deciduous forests. *Ecological Informatics*.

Thorp, K.R., Wang, G., Bronson, K.F., Badaruddin, M., and Mon, J. (2017). Hyperspectral data mining to identify relevant canopy spectral features for estimating durum wheat growth, nitrogen status, and grain yield. *Computers & Electronics in Agriculture* 136**,** 1-12.

Tong, A., and He, Y. (2017). Estimating and mapping chlorophyll content for a heterogeneous grassland: Comparing prediction power of a suite of vegetation indices across scales between years. *Isprs Journal of Photogrammetry and Remote Sensing* 126**,** 146-167.

Wang, L., Pu, H., and Sun, D. (2016). Estimation of chlorophyll-a concentration of different seasons in outdoor ponds using hyperspectral imaging. *Talanta* 147**,** 422-429.

Wang, Z., Skidmore, A.K., Wang, T., Darvishzadeh, R., Heiden, U., Heurich, M., Latifi, H., and Hearne, J. (2017). Canopy foliar nitrogen retrieved from airborne hyperspectral imagery by correcting for canopy structure effects. *International Journal of Applied Earth Observation and Geoinformation* 54**,** 84-94.

Wu, Q., Wang, J., Wang, C., and Xu, T. (2016). Study on the optimal algorithm prediction of corn leaf component information based on hyperspectral imaging. *Infrared Physics & Technology* 78**,** 66-71.

Yi, Q., Jiapaer, G., Chen, J., Bao, A., and Wang, F. (2014). Different units of measurement of carotenoids estimation in cotton using hyperspectral indices and partial least square regression. *Isprs Journal of Photogrammetry and Remote Sensing* 91**,** 72-84.

Yu, K., Lenzwiedemann, V., Chen, X., and Bareth, G. (2014). Estimating leaf chlorophyll of barley at different growth stages using spectral indices to reduce soil background and canopy structure effects. *Isprs Journal of Photogrammetry and Remote Sensing* 97**,** 58-77.

Zhang, C., Wang, Q., Liu, F., He, Y., and Xiao, Y. (2017). Rapid and non-destructive measurement of spinach pigments content during storage using hyperspectral imaging with chemometrics. *Measurement* 97**,** 149-155.

Zhou, L., Roberts, D.A., Ma, W., Zhang, H., and Tang, L. (2014). Estimation of higher chlorophylla concentrations using field spectral measurement and HJ-1A hyperspectral satellite data in Dianshan Lake, China. *Isprs Journal of Photogrammetry and Remote Sensing* 88**,** 41-47.

Zhou, X., Huang, W., Kong, W., Ye, H., Luo, J., and Chen, P. (2016). Remote estimation of canopy nitrogen content in winter wheat using airborne hyperspectral reflectance measurements. *Advances in Space Research* 58**,** 1627-1637.

Zou, X., Shi, J., Hao, L., Zhao, J., Mao, H., Chen, Z., Li, Y., and Holmes, M. (2011). In vivo noninvasive detection of chlorophyll distribution in cucumber (Cucumis sativus) leaves by indices based on hyperspectral imaging. *Analytica Chimica Acta* 706**,** 105-112.
