# Supplementary material for: Accurate Digitization of the Chlorophyll Distribution of Individual Rice Leaves Using Hyperspectral Imaging and an Integrated Image Analysis Pipeline
Source: Front Plant Sci. 2017 Jul 25;8:1238. doi: 10.3389/fpls.2017.01238 (PMC5524744; doi:10.3389/fpls.2017.01238)
Supplement: Supplementary Table 2 — Information about the 90 rice accessions and SPAD value. [file Table2.DOCX]

Supplementary Table 2 Information about the 90 rice accessions and SPAD value.

| Number | Accession | Nitrogen (mg/g) | SPAD |
| --- | --- | --- | --- |
| 1 | BASMATI 385 | 38.6362 | 44.6 |
| 2 | B805D-MR-16-8-3 | 37.3995 | 48.7 |
| 3 | BLUE STICK | 41.9836 | 45.5 |
| 4 | Ximaxian | 41.2256 | 45.3 |
| 5 | Ma ba you zhan | 36.3960 | 40.6 |
| 6 | TAINUNG 45 | 30.6594 | 38.7 |
| 7 | Jinbaoyin | 30.9978 | 37.4 |
| 8 | Manawthukha | 36.7206 | 38.7 |
| 9 | Hongmisandan | 42.0478 | 45.2 |
| 10 | X22 | 42.3877 | 46.5 |
| 11 | Tsao wan ching | 40.2403 | 44 |
| 12 | Guantuibaihe | 42.7417 | 45.5 |
| 13 | Mowanggunei-1 | 34.0878 | 37.8 |
| 14 | SORNAVARI | 31.7951 | 38.9 |
| 15 | Sugeng2hao | 44.4619 | 46 |
| 16 | Laohuzhong | 40.8045 | 47.2 |
| 17 | Xiaohonggu | 33.9797 | 38.4 |
| 18 | Babaili | 38.8121 | 47.7 |
| 19 | Wuzidui | 40.0279 | 42.7 |
| 20 | Biwusheng | 30.4446 | 40.6 |
| 21 | ASD16 | 45.0288 | 45.8 |
| 22 | SADAJIRA-19-303 | 32.3674 | 40.4 |
| 23 | Babaomi | 28.4492 | 31.9 |
| 24 | Tainong67 | 42.6434 | 42.1 |
| 25 | Dom Sufid | 41.0778 | 44.1 |
| 26 | Baikezaohe | 41.3378 | 43.1 |
| 27 | CPSLO 17 | 36.0603 | 44.2 |
| 28 | Diantun502 | 41.3178 | 40.4 |
| 29 | TB154E-TB-2 | 27.7227 | 33.6 |
| 30 | Gallawa | 36.9646 | 40.8 |
| 31 | Gu154 | 32.0870 | 37.5 |
| 32 | PeiC122 | 31.5437 | 38.2 |
| 33 | Momi | 34.6259 | 38.6 |
| 34 | R 75 | 29.7538 | 38.2 |
| 35 | PR 116 | 31.0691 | 38.8 |
| 36 | Qitougu | 35.2967 | 36.4 |
| 37 | MILYANG 23 | 32.2424 | 43.9 |
| 38 | Zhengxian232 | 36.1300 | 40 |
| 39 | Toga | 42.3331 | 42.7 |
| 40 | OM1723 | 28.0213 | 39.6 |
| 41 | Sholay | 35.6599 | 42.7 |
| 42 | CIGEULIS | 38.3379 | 46.1 |
| 43 | Shanjiugu | 42.0210 | 46.6 |
| 44 | WC 2811 | 35.1362 | 39.6 |
| 45 | LABELLE | 36.0820 | 42.9 |
| 46 | Latisai1 | 32.9243 | 40 |
| 47 | Xiangnuo-1 | 39.6736 | 49.9 |
| 48 | Jumli dhan | 35.2970 | 42.1 |
| 49 | Guihuahuang | 47.3497 | 46.8 |
| 50 | WH105 | 42.2109 | 49.2 |
| 51 | WAB462-10-3-1 | 47.0739 | 49.6 |
| 52 | IARI 6621 | 41.4636 | 45.1 |
| 53 | 80B | 33.1680 | 38.9 |
| 54 | Moisdol | 44.3797 | 49.1 |
| 55 | 99216 | 37.5411 | 44.5 |
| 56 | AUS 373 | 30.6905 | 39.3 |
| 57 | Heiheaihui | 35.6719 | 45.7 |
| 58 | Karnal Local | 34.3977 | 38.3 |
| 59 | CR203 | 32.0178 | 44.1 |
| 60 | Gayabyeo | 40.4407 | 47 |
| 61 | NEW BONNET | 40.0511 | 46.1 |
| 62 | Bawangbian1 | 42.4821 | 44.9 |
| 63 | Taizhongxianxuan2 | 30.4584 | 41 |
| 64 | C 5560 | 37.4895 | 40.9 |
| 65 | IARI 6626 | 44.0223 | 46.7 |
| 66 | TD 70 | 33.6090 | 39.8 |
| 67 | Aituogu151 | 42.3103 | 43.9 |
| 68 | PSB RC 28 | 41.2686 | 42.6 |
| 69 | PATNAI 6 | 35.9560 | 42.1 |
| 70 | ASD18 | 35.7343 | 39.9 |
| 71 | 452 | 41.1590 | 47.5 |
| 72 | menjiading2 | 40.7005 | 44.2 |
| 73 | TCHAMPA | 41.4469 | 40 |
| 74 | Mengguandamagu | 41.7477 | 45.2 |
| 75 | ASWINA 330 | 44.8986 | 39.3 |
| 76 | Chikenuo | 31.1593 | 38.6 |
| 77 | Won Son Zo No. 11 | 46.4266 | 47.4 |
| 78 | R 67 | 42.0949 | 49.6 |
| 79 | TEQING | 31.4721 | 40 |
| 80 | Xugunuo | 42.0919 | 44.8 |
| 81 | Xiaobaimi | 36.9699 | 44.2 |
| 82 | CAROLINO 164 | 44.0912 | 48.5 |
| 83 | Zhong413 | 43.6442 | 41.5 |
| 84 | RUBIO | 46.0583 | 45.5 |
| 85 | Cunsanli | 45.5245 | 46.3 |
| 86 | Muguanuo-2 | 53.2565 | 48.8 |
| 87 | A 152 | 24.2454 | 34.7 |
| 88 | Maweinian | 40.8288 | 47.1 |
| 89 | Dianrui409B | 29.6286 | 39.2 |
| 90 | Muxiqiu | 31.2861 | 38.1 |
