# Supplementary material for: Accurate Digitization of the Chlorophyll Distribution of Individual Rice Leaves Using Hyperspectral Imaging and an Integrated Image Analysis Pipeline
Source: Front Plant Sci. 2017 Jul 25;8:1238. doi: 10.3389/fpls.2017.01238 (PMC5524744; doi:10.3389/fpls.2017.01238)
Supplement: Supplementary Table 3 — Distribution of the pigments at the two stages. [file Table3.DOCX]

Supplementary Table 3 Distribution of the pigments at the two stages.

| Stage | Pigment | Number | Average(*mg/m²*) | standard deviation  (*mg/m²*) | CV |
| --- | --- | --- | --- | --- | --- |
| Tillering stage | Chlorophyll a | 427 | 294.35 | 92.19 | 31.32% |
|  | Chlorophyll b | 427 | 78.11 | 26.91 | 34.44% |
|  | Total chlorophyll | 427 | 372.47 | 117.94 | 31.66% |
|  | Carotenoid | 427 | 72.08 | 19.12 | 26.53% |
| Heading stage | Chlorophyll a | 149 | 283.76 | 67.24 | 23.70% |
|  | Chlorophyll b | 149 | 75.84 | 20.13 | 26.54% |
|  | Total chlorophyll | 149 | 359.59 | 86.79 | 24.14% |
|  | Carotenoid | 149 | 62.64 | 14.55 | 23.22% |
