# Supplementary material for: Accurate Digitization of the Chlorophyll Distribution of Individual Rice Leaves Using Hyperspectral Imaging and an Integrated Image Analysis Pipeline
Source: Front Plant Sci. 2017 Jul 25;8:1238. doi: 10.3389/fpls.2017.01238 (PMC5524744; doi:10.3389/fpls.2017.01238)
Supplement: Supplementary Table 4 — Correlation coefficient (r) between the pigments at the two stages. [file Table4.DOCX]

Supplementary Table 4 Correlation coefficient (r) between the pigments at the two stages.

|  | Pigment | Chlorophyll a | Chlorophyll b | Total chlorophyll | Carotenoid |
| --- | --- | --- | --- | --- | --- |
| Tillering stage | Chlorophyll a | 1 |  |  |  |
|  | Chlorophyll b | 0.9459 | 1 |  |  |
|  | Total chlorophyll | 0.9973 | 0.9673 | 1 |  |
|  | Carotenoid | 0.9542 | 0.9045 | 0.9520 | 1 |
| Heading stage | Chlorophyll a | 1 |  |  |  |
|  | Chlorophyll b | 0.9140 | 1 |  |  |
|  | Total chlorophyll | 0.9947 | 0.9508 | 1 |  |
|  | Carotenoid | 0.9549 | 0.8839 | 0.9527 | 1 |
