# Supplementary material for: Accurate Digitization of the Chlorophyll Distribution of Individual Rice Leaves Using Hyperspectral Imaging and an Integrated Image Analysis Pipeline
Source: Front Plant Sci. 2017 Jul 25;8:1238. doi: 10.3389/fpls.2017.01238 (PMC5524744; doi:10.3389/fpls.2017.01238)
Supplement: Supplementary Table 5 — Spectral index based on spectral position and area. [file Table5.DOCX]

Supplementary Table 5 Spectral index based on spectral position and area.

| Hyperspectral indices | Definitions |
| --- | --- |
| CPR_1_ | The normalized ratio of the *CPR_14_* and *CPR_17_*([Huang et al., 2010](#_ENREF_1)) |
| CPR_2_ | The normalized ratio of the *CPR_14_* and *CPR_20_*([Huang et al., 2010](#_ENREF_1)) |
| CPR_3_ | The ratio of the *CPR_14_* and *CPR_17_*([Huang et al., 2010](#_ENREF_1)) |
| CPR_4_ | The ratio of the*CPR_14_* and *CPR_20_*([Huang et al., 2010](#_ENREF_1)) |
| CPR_5_ | The normalized ratio of the *CPR_10_* and *CPR_8_*([Huang et al., 2010](#_ENREF_1)) |
| CPR_6_ | The ratio of the *CPR_10_* and *CPR_8_*([Huang et al., 2010](#_ENREF_1)) |
| CPR_7_ | The red valley position of the *R_i_* |
| CPR_8_ | The red valley reflectance of the *R_i_* |
| CPR_9_ | The green peak position of the *R_i_* |
| CPR_10_ | The green peak reflectance of the *R_i_* |
| CPR_11_ | The green peak area of the *R_i_* |
| CPR_12_ | The red edge position of the *R_i_* |
| CPR_13_ | The red edge amplitude of the *R_i_* |
| CPR_14_ | The red edge area of the *R_i_* |
| CPR_15_ | The yellow edge position of the *R_i_* |
| CPR_16_ | The yellow edge amplitude of the *R_i_* |
| CPR_17_ | The yellow edge area of the *R_i_* |
| CPR_18_ | The blue edge position of the *R_i_* |
| CPR_19_ | The blue edge amplitude of the *R_i_* |
| CPR_20_ | The blue edge area of the *R_i_* |

Huang, J., Wang, F., and Wang, X. (2010). "Hyperspectral experiment for paddy rice remote sensing". Zhejiang University Press).
