# Supplementary material for: Accurate Digitization of the Chlorophyll Distribution of Individual Rice Leaves Using Hyperspectral Imaging and an Integrated Image Analysis Pipeline
Source: Front Plant Sci. 2017 Jul 25;8:1238. doi: 10.3389/fpls.2017.01238 (PMC5524744; doi:10.3389/fpls.2017.01238)
Supplement: Supplementary Table 6 — Published spectral indices∗. [file Table6.DOCX]

**Supplementary Table 6** Published spectral indices*.

| Index | Reference | Index | Reference |
| --- | --- | --- | --- |
| $\frac{R_{415}-R_{435}}{R_{415}+R_{435}}$ | ([Barnes et al., 1992](#_ENREF_2)) | $\frac{D_{725}}{D_{702}} \frac{D_{715}}{D_{702}}$ | ([Kochubey and Kazantsev, 2007](#_ENREF_25)) |
| $\frac{R_{550}}{R_{800}}$ | ([Aoki et al., 1981](#_ENREF_1)) | $\sum_{i=626}^{795} D_{i}$ | ([Elvidge and Chen, 1995](#_ENREF_12)) |
| $\frac{R_{750}-R_{705}}{R_{750}+R_{705}}$ $\int_{705}^{750} (R_{\lambda}/R_{705}-1)d\lambda$   | ([Gamon and Surfus, 1999](#_ENREF_14);[Richardson et al., 2002](#_ENREF_33)) | $R_{880}-R_{550}$ $R_{880}/R_{550}$   | ([Buschmann and Nagel, 1993](#_ENREF_7)) |
| $R_{800}-R_{675}$ | ([Jordan, 1969](#_ENREF_24)) | $R_{750}/R_{700}$ | ([Gitelson et al., 1996b](#_ENREF_20)) |
| $\frac{D_{722}-D_{700}}{D_{722}+D_{700}}$ $\sum dRE/\sum db$ $(\sum dRE-\sum db)/(\sum dRE+\sum db)$ $dRE/dB$ $(dRE-dB)/(dRE+dB)$         | ([Xue and Yang, 2009](#_ENREF_41)) | $R_{672}/(R_{550}\times R_{708})$ $R_{675}$ $R_{675}/R_{700}$ $D_{715}/D_{705}$ $R_{675}/R_{700}$ $R_{675}/(R_{700}\times R_{650})$         | ([Chappelle et al., 1992](#_ENREF_9)) |
| $R_{700}/R_{670}$ | ([McMurtrey et al., 1994](#_ENREF_29)) | $R_{554}/R_{677}$ | ([Smith et al., 1995](#_ENREF_37)) |
| $\frac{\mathrm{R}_{750}/\mathrm{R}_{705}-1}{\sqrt{\mathrm{R}_{750}/\mathrm{R}_{705}+1}}$ $[(R_{750}-R_{705})-0.2(R_{750}-R_{550})](\frac{R_{750}}{R_{705}})$ $\frac{3[(R_{750}-R_{705})-0.2(R_{750}-R_{550})(R_{750}/R_{705})]}{(1+0.16)(R_{750}-R_{705})/(R_{750}+R_{705}+0.16)}$ $\frac{[(R_{750}-R_{705})-0.2(R_{750}-R_{550})](R_{750}/R_{705})}{(1+0.16)(R_{750}-R_{705})/(R_{750}+R_{705}+0.16)}$       | ([Wu et al., 2008](#_ENREF_40)) | $R_{710}/R_{760}$ $(R_{780}-R_{710})/(R_{780}-R_{680})$ $(R_{750}-R_{705})/(R_{750}+R_{705})$ $(R_{680}-R_{430})/(R_{680}+R_{430})$ $R_{860}/(R_{550}\times R_{708})$         | ([Zou et al., 2011](#_ENREF_45)) |
| $R_{550}$ | ([Thomas and Gausman, 1977](#_ENREF_38);[Jacquemoud and Baret, 1990](#_ENREF_23)) | $R_{745}/R_{675}$ | ([Birth and McVey, 1968](#_ENREF_3)) |
| $(R_{800}-R_{680})/(R_{800}+R_{680})$ | ([Rouse et al., 1974](#_ENREF_34)) | $R_{750}/R_{700}$ $R_{750}/R_{550}$ | ([Gitelson and Merzlyak, 1997](#_ENREF_19)) |
| $(R_{780}-R_{710})/(R_{780}-R_{680})$ $(R_{542}-R_{min\_red})/(R_{750}-R_{min\_red})$   | ([Maccioni et al., 2001](#_ENREF_28)) | $R_{750}/R_{700}$ $R_{750}/R_{550}$   | ([Lichtenthaler et al., 1996](#_ENREF_27)) |
| $(R_{678}-R_{500})/R_{750}$ | ([Merzlyak et al., 1999](#_ENREF_30)) | $D(log(1/R_{737}))$ | ([Yoder and Pettigrew, 1995](#_ENREF_42)) |
| $(R_{800}-R_{680})/(R_{800}+R_{680})$ $(R_{800}-R_{635})/(R_{800}+R_{635})$ $R_{800}/R_{680}$ $R_{800}/R_{650}$     | ([Blackburn, 1998a](#_ENREF_4);[b](#_ENREF_5)) | $R_{695}/R_{670}$ $R_{695}/R_{760}$ $R_{710}/R_{760}$ $R_{695}/R_{420}$ $R_{605}/R_{760}$ $R_{550}$     | ([Carter, 1994](#_ENREF_8)) |
| $R_{430}/R_{680}$ | ([Horler et al., 1983](#_ENREF_21)) | $\sum_{i=680}^{780} D_{i}$ | ([Filella and Penuelas, 1994](#_ENREF_13)) |
| $D_{715}/D_{705}$ $R_{740}/R_{720}$ $(R_{734}-R_{747})/\text{(}R_{715}+R_{726})$ $R_{740}/R_{720}$     | ([Vogelmann et al., 1993](#_ENREF_39)) | $R_{430}/R_{680}$ $dRE/dG$ $(R_{680}-R_{430})/(R_{680}+R_{430})$ $(dRE-dG)/(dRE+dG)$     | ([Peñuelas et al., 1994](#_ENREF_31)) |
| $(R_{750}-R_{445})/(R_{705}-R_{445})$ $(R_{750}-R_{705})/(R_{750}+R_{705}-2R_{445})$   | ([Sims and Gamon, 2002](#_ENREF_36)) | $D_{721}$ $\sum D_{680-780}$ $D(D_{728})$ $D(Log1/R_{737})$   | ([Blackburn and Pitman, 1999](#_ENREF_6)) |
| $R_{672}/(R_{550}\times R_{708})$ $R_{860}/(R_{550}\times R_{708})$   | ([Datt, 1998](#_ENREF_10)) | $(R_{850}-R_{710})/(R_{850}-R_{680})$ $D_{754}/D_{704}$   | ([Datt, 1999](#_ENREF_11)) |
| $R_{630}/R_{680}$ $R_{600}/R_{680}$ | ([Schlemmer et al., 2005](#_ENREF_35)) | $\sum_{i=400}^{700} R_{i}$ | ([Zarco et al., 2001](#_ENREF_44)) |
| $(R_{800}-R_{445})/(R_{800}-R_{680})$ | ([Penuelas et al., 1995](#_ENREF_32)) | $1/R_{700}$ | ([Gitelson et al., 1999](#_ENREF_15)) |
| $R_{750}/R_{550}$ $R{}_{750}/R_{700}$ | ([Gitelson and Merzlyak, 1996](#_ENREF_18)) | $(R_{780}-R_{550})/(R_{780}+R_{550})$ | ([Gitelson et al., 1996a](#_ENREF_16)) |
| $R_{750}/R_{700}$ $R_{750}/R_{550}$ $(R_{750}-R_{705})/(R_{750}+R_{705})$ $\int_{705}^{750} (R_{\lambda}/R_{705}-1)d\lambda$     | ([Gitelson and Merzlyak, 1994](#_ENREF_17)) | $D_{705}/D_{702}$ $D_{705}/D_{722}$ $D_{715}/D_{705}$ $\sum dRE/\sum dB$     | ([Zarco et al., 2002](#_ENREF_43)) |
| $(R_{728}-R_{434})/(R_{720}-R_{434})$ $(R_{728}-R_{720})/(R_{728}+R_{720}-2R_{434})$ $(D_{722}-D_{700})/(D_{722}+D_{700})$ $(D_{722}-D_{502})/(D_{700}-D_{502})$ $(D_{722}-D_{700})/(D_{722}+D_{700}-2D_{502})$         | ([Le Maire et al., 2004](#_ENREF_26)) |  |  |

* D is d(R).

**Supplementary References**

Aoki, M., Yabuki, K., and Totsuka, T. (1981). An evaluation of chlorophyll content of leaves based on the spectral reflectivity in several plants. *Research Reports of the National Institute of Environmental Studies of Japan* 66**,** 125-130.

Barnes, J., Balaguer, L., Manrique, E., Elvira, S., and Davison, A. (1992). A reappraisal of the use of DMSO for the extraction and determination of chlorophylls a and b in lichens and higher plants. *Environmental and Experimental Botany* 32**,** 85-100.

Birth, G., and Mcvey, G. (1968). Measuring the color of growing turf with a reflectance spectrophotometer. *Agronomy Journal* 60**,** 640-643.

Blackburn, G. (1998a). Quantifying chlorophylls and caroteniods at leaf and canopy scales: An evaluation of some hyperspectral approaches. *Remote sensing of environment* 66**,** 273-285.

Blackburn, G. (1998b). Spectral indices for estimating photosynthetic pigment concentrations: a test using senescent tree leaves. *International Journal of Remote Sensing* 19**,** 657-675.

Blackburn, G., and Pitman, J. (1999). Biophysical controls on the directional spectral reflectance properties of bracken (Pteridium aquilinum) canopies: results of a field experiment. *International Journal of Remote Sensing* 20**,** 2265-2282.

Buschmann, C., and Nagel, E. (1993). In vivo spectroscopy and internal optics of leaves as basis for remote sensing of vegetation. *International Journal of Remote Sensing* 14**,** 711-722.

Carter, G. (1994). Ratios of leaf reflectances in narrow wavebands as indicators of plant stress. *Remote sensing* 15**,** 697-703.

Chappelle, E., Kim, M., and Mcmurtrey, J. (1992). Ratio analysis of reflectance spectra (RARS): an algorithm for the remote estimation of the concentrations of chlorophyll a, chlorophyll b, and carotenoids in soybean leaves. *Remote Sensing of Environment* 39**,** 239-247.

Datt, B. (1998). Remote Sensing of Chlorophyll a, Chlorophyll b, Chlorophyll a+b, and Total Carotenoid Content in Eucalyptus Leaves. *Remote Sensing of Environment* 66**,** 111-121.

Datt, B. (1999). Visible/near infrared reflectance and chlorophyll content in Eucalyptus leaves. *International Journal of Remote Sensing* 20**,** 2741-2759.

Elvidge, C., and Chen, Z. (1995). Comparison of broad-band and narrow-band red and near-infrared vegetation indices. *Remote sensing of environment* 54**,** 38-48.

Filella, I., and Penuelas, J. (1994). The red edge position and shape as indicators of plant chlorophyll content, biomass and hydric status. *International Journal of Remote Sensing* 15**,** 1459-1470.

Gamon, J., and Surfus, J. (1999). Assessing leaf pigment content and activity with a reflectometer. *New Phytologist* 143**,** 105-117.

Gitelson, A., Buschmann, C., and Lichtenthaler, H. (1999). The Chlorophyll Fluorescence Ratio F735/F 700 as an Accurate Measure of the Chlorophyll Content in Plants. *Remote Sensing of Environment* 69**,** 296-302.

Gitelson, A., Kaufman, Y., and Merzlyak, M. (1996a). Use of a green channel in remote sensing of global vegetation from EOS-MODIS. *Remote Sensing of Environment* 58**,** 289-298.

Gitelson, A., and Merzlyak, M. (1994). Spectral reflectance changes associated with autumn senescence of Aesculus hippocastanum L. and Acer platanoides L. leaves. Spectral features and relation to chlorophyll estimation. *Journal of Plant Physiology* 143**,** 286-292.

Gitelson, A., and Merzlyak, M. (1996). Signature analysis of leaf reflectance spectra: algorithm development for remote sensing of chlorophyll. *Journal of plant physiology* 148**,** 494-500.

Gitelson, A., and Merzlyak, M. (1997). Remote estimation of chlorophyll content in higher plant leaves. *International Journal of Remote Sensing* 18**,** 2691-2697.

Gitelson, A., Merzlyak, M., and Lichtenthaler, H. (1996b). Detection of red edge position and chlorophyll content by reflectance measurements near 700 nm. *Journal of Plant Physiology* 148**,** 501-508.

Horler, D., Dockray, M., Barber, J., and Barringer, A. (1983). Red edge measurements for remotely sensing plant chlorophyll content. *Advances in Space Research* 3**,** 273-277.

Huang, J., Wang, F., and Wang, X. (2010). "Hyperspectral experiment for paddy rice remote sensing". Zhejiang University Press).

Jacquemoud, S., and Baret, F. (1990). PROSPECT: A model of leaf optical properties spectra. *Remote sensing of environment* 34**,** 75-91.

Jordan, C. (1969). Derivation of leaf-area index from quality of light on the forest floor. *Ecology***,** 663-666.

Kochubey, S.M., and Kazantsev, T.A. (2007). Changes in the first derivatives of leaf reflectance spectra of various plants induced by variations of chlorophyll content. *Journal of Plant Physiology* 164**,** 1648-1655.

Le Maire, G., Francois, C., and Dufrene, E. (2004). Towards universal broad leaf chlorophyll indices using PROSPECT simulated database and hyperspectral reflectance measurements. *Remote sensing of environment* 89**,** 1-28.

Lichtenthaler, H., Gitelson, A., and Lang, M. (1996). Non-destructive determination of chlorophyll content of leaves of a green and an aurea mutant of tobacco by reflectance measurements. *Journal of Plant Physiology* 148**,** 483-493.

Maccioni, A., Agati, G., and Mazzinghi, P. (2001). New vegetation indices for remote measurement of chlorophylls based on leaf directional reflectance spectra. *Journal of Photochemistry and Photobiology B: Biology* 61**,** 52-61.

Mcmurtrey, J., Chappelle, E., Kim, M., Meisinger, J., and Corp, L. (1994). Distinguishing nitrogen fertilization levels in field corn (Zea mays L.) with actively induced fluorescence and passive reflectance measurements. *Remote sensing of environment* 47**,** 36-44.

Merzlyak, M., Gitelson, A., Chivkunova, O., and Rakitin, V. (1999). Non‐destructive optical detection of pigment changes during leaf senescence and fruit ripening. *Physiologia plantarum* 106**,** 135-141.

Peñuelas, J., Gamon, J., Fredeen, A., Merino, J., and Field, C. (1994). Reflectance indices associated with physiological changes in nitrogen-and water-limited sunflower leaves. *Remote Sensing of Environment* 48**,** 135-146.

Penuelas, J., Baret, F., and Filella, I. (1995). Semi-empirical indices to assess carotenoids/chlorophyll a ratio from leaf spectral reflectance. *Photosynthetica* 31**,** 221-230.

Richardson, A., Duigan, S., and Berlyn, G. (2002). An evaluation of noninvasive methods to estimate foliar chlorophyll content. *New Phytologist* 153**,** 185-194.

Rouse , J., Haas, R., Schell, J., and Deering, D. (1974). Monitoring vegetation systems in the Great Plains with ERTS. *NASA special publication* 351**,** 309.

Schlemmer, M., Francis, D., Shanahan, J., and Schepers, J. (2005). Remotely measuring chlorophyll content in corn leaves with differing nitrogen levels and relative water content. *Agronomy Journal* 97**,** 106-112.

Sims, D., and Gamon, J. (2002). Relationships between leaf pigment content and spectral reflectance across a wide range of species, leaf structures and developmental stages. *Remote sensing of environment* 81**,** 337-354.

Smith, R., Adams, J., Stephens, D., and Hick, P. (1995). Forecasting wheat yield in a Mediterranean-type environment from the NOAA satellite. *Crop and Pasture Science* 46**,** 113-125.

Thomas, J., and Gausman, H. (1977). Leaf reflectance vs. leaf chlorophyll and carotenoid concentrations for eight crops. *Agronomy journal* 69**,** 799-802.

Vogelmann, J., Rock, B., and Moss, D. (1993). Red edge spectral measurements from sugar maple leaves. *TitleREMOTE SENSING* 14**,** 1563-1575.

Wu, C., Niu, Z., Tang, Q., and Huang, W. (2008). Estimating chlorophyll content from hyperspectral vegetation indices: Modeling and validation. *agricultural and forest meteorology* 148**,** 1230-1241.

Xue, L., and Yang, L. (2009). Deriving leaf chlorophyll content of green-leafy vegetables from hyperspectral reflectance. *ISPRS Journal of Photogrammetry and Remote Sensing* 64**,** 97-106.

Yoder, B., and Pettigrew, R. (1995). Predicting nitrogen and chlorophyll content and concentrations from reflectance spectra (400–2500 nm) at leaf and canopy scales. *Remote sensing of environment* 53**,** 199-211.

Zarco, P., Miller, J., Mohammed, G., Noland, T., and Sampson, P. (2002). Vegetation Stress Detection through Chlorophyll+ Estimation and Fluorescence Effects on Hyperspectral Imagery. *Journal of environmental quality* 31**,** 1433-1441.

Zarco, P., Miller, J., Noland, T., Mohammed, G., and Sampson, P. (2001). Scaling-up and model inversion methods with narrowband optical indices for chlorophyll content estimation in closed forest canopies with hyperspectral data. *Geoscience and Remote Sensing, IEEE Transactions on* 39**,** 1491-1507.

Zou, X., Shi, J., Hao, L., Zhao, J., Mao, H., Chen, Z., Li, Y., and Mel, H. (2011). In vivo noninvasive detection of chlorophyll distribution in cucumber (Cucumis sativus) leaves by indices based on hyperspectral imaging. *Analytica chimica acta* 706**,** 105-112.
