# Supplementary material for: Accurate Digitization of the Chlorophyll Distribution of Individual Rice Leaves Using Hyperspectral Imaging and an Integrated Image Analysis Pipeline
Source: Front Plant Sci. 2017 Jul 25;8:1238. doi: 10.3389/fpls.2017.01238 (PMC5524744; doi:10.3389/fpls.2017.01238)
Supplement: Supplementary Table 7 — Comparison of performance between models using all of the indices and models using original indices. [file Table7.DOCX]

Supplementary Table 7 Comparison of performance between models using all of the indices and models using original indices.

| Stage | Pigment | Modeling using all of the indices | | | | Modeling using original indices | | |
| --- | --- | --- | --- | --- | --- | --- | --- | --- |
|  |  | Variable  numbers | R² | $R_{adj}^{2}$ | RMSE  (*mg/m²*) | R² | $R_{adj}^{2}$ | RMSE  (*mg/m²*) |
| Tillering stage | Chlorophyll a | 1 | 0.896 | 0.896 | 29.705 | 0.845 | 0.845 | 36.326 |
|  |  | 2 | 0.924 | 0.924 | 25.433 | 0.901 | 0.900 | 29.138 |
|  |  | 3 | 0.934 | 0.933 | 23.781 | 0.912 | 0.912 | 27.425 |
|  |  | 4 | 0.942 | 0.941 | 22.781 | 0.919 | 0.918 | 26.421 |
|  | Chlorophyll b | 1 | 0.799 | 0.799 | 12.065 | 0.785 | 0.785 | 12.479 |
|  |  | 2 | 0.836 | 0.835 | 10.919 | 0.809 | 0.809 | 11.773 |
|  |  | 3 | 0.857 | 0.856 | 10.207 | 0.822 | 0.821 | 11.397 |
|  |  | 4 | 0.865 | 0.864 | 9.927 | 0.830 | 0.828 | 11.149 |
|  | Chlorophyll | 1 | 0.876 | 0.876 | 41.583 | 0.846 | 0.846 | 46.328 |
|  |  | 2 | 0.914 | 0.913 | 34.729 | 0.894 | 0.894 | 38.441 |
|  |  | 3 | 0.926 | 0.925 | 32.212 | 0.907 | 0.906 | 36.143 |
|  |  | 4 | 0.932 | 0.931 | 30.879 | 0.913 | 0.912 | 34.927 |
|  | Carotenoid | 1 | 0.807 | 0.807 | 8.409 | 0.719 | 0.719 | 10.144 |
|  |  | 2 | 0.838 | 0.837 | 7.711 | 0.775 | 0.774 | 9.090 |
|  |  | 3 | 0.851 | 0.850 | 7.411 | 0.793 | 0.791 | 8.740 |
|  |  | 4 | 0.865 | 0.863 | 7.069 | 0.805 | 0.804 | 8.474 |
| Heading stage | Chlorophyll a | 1 | 0.699 | 0.697 | 36.987 | 0.699 | 0.697 | 36.987 |
|  |  | 2 | 0.777 | 0.774 | 31.979 | 0.777 | 0.774 | 31.979 |
|  |  | 3 | 0.816 | 0.812 | 29.141 | 0.783 | 0.778 | 31.654 |
|  |  | 4 | 0.838 | 0.833 | 27.467 | 0.805 | 0.800 | 30.084 |
|  | Chlorophyll b | 1 | 0.711 | 0.709 | 10.854 | 0.698 | 0.696 | 11.105 |
|  |  | 2 | 0.760 | 0.757 | 9.925 | 0.754 | 0.750 | 10.062 |
|  |  | 3 | 0.800 | 0.795 | 9.105 | - | - | - |
|  |  | 4 | 0.836 | 0.831 | 8.265 | - | - | - |
|  | Chlorophyll | 1 | 0.708 | 0.706 | 47.042 | 0.700 | 0.698 | 47.665 |
|  |  | 2 | 0.782 | 0.779 | 40.783 | 0.780 | 0.777 | 41.006 |
|  |  | 3 | 0.821 | 0.817 | 37.111 | 0.787 | 0.783 | 40.468 |
|  |  | 4 | 0.856 | 0.852 | 33.429 | 0.809 | 0.804 | 38.460 |
|  | Carotenoid | 1 | 0.612 | 0.610 | 9.091 | 0.612 | 0.610 | 9.091 |
|  |  | 2 | 0.672 | 0.667 | 8.392 | 0.661 | 0.656 | 8.530 |
|  |  | 3 | 0.747 | 0.742 | 7.393 | 0.672 | 0.665 | 8.423 |
|  |  | 4 | 0.776 | 0.770 | 6.979 | 0.718 | 0.711 | 7.828 |
