# Supplementary material for: Accurate Digitization of the Chlorophyll Distribution of Individual Rice Leaves Using Hyperspectral Imaging and an Integrated Image Analysis Pipeline
Source: Front Plant Sci. 2017 Jul 25;8:1238. doi: 10.3389/fpls.2017.01238 (PMC5524744; doi:10.3389/fpls.2017.01238)
Supplement: Supplementary Table 8 — Details of the multiple-variable models for 4 pigments. [file Table8.DOCX]

Supplementary Table 8 Details of the multiple-variable models for 4 pigments.

| Stage | Pigment | Variable  numbers | R² | $R_{adj}^{2}$ | MAPE | RMSE(*mg/m²*) | 5-fold cross validation | | | | |
| --- | --- | --- | --- | --- | --- | --- | --- | --- | --- | --- | --- |
|  |  |  |  |  |  |  | Modeling | | | Validation | |
|  |  |  |  |  |  |  | R² | MAPE | RMSE(*mg/m²*) | MAPE | RMSE(*mg/m²*) |
| Tillering stage | Chlorophyll a | 1 | 0.896 | 0.896 | 8.14% | 29.705 | 0.893 | 8.15% | 29.623 | 8.40% | 30.014 |
|  |  | 2 | 0.924 | 0.924 | 6.69% | 25.433 | 0.923 | 6.70% | 25.320 | 6.83% | 26.206 |
|  |  | 3 | 0.934 | 0.933 | 6.37% | 23.781 | 0.933 | 6.36% | 23.654 | 6.39% | 24.072 |
|  |  | 4 | 0.942 | 0.941 | 6.37% | 22.781 | 0.945 | 6.026% | 22.233 | 6.17% | 22.785 |
|  | Chlorophyll b | 1 | 0.799 | 0.799 | 13.51% | 12.065 | 0.807 | 13.49% | 12.034 | 13.56% | 12.072 |
|  |  | 2 | 0.836 | 0.835 | 12.39% | 10.919 | 0.838 | 12.39% | 10.873 | 12.22% | 11.074 |
|  |  | 3 | 0.857 | 0.856 | 11.23% | 10.207 | 0.861 | 11.22% | 10.146 | 11.26% | 10.613 |
|  |  | 4 | 0.865 | 0.864 | 11.00% | 9.927 | 0.864 | 10.988% | 9.858 | 11.06% | 10.162 |
|  | Total chlorophyll | 1 | 0.876 | 0.876 | 9.34% | 41.583 | 0.880 | 9.32% | 41.139 | 9.88% | 42.736 |
|  |  | 2 | 0.914 | 0.913 | 7.76% | 34.729 | 0.912 | 7.72% | 34.523 | 9.08% | 37.534 |
|  |  | 3 | 0.926 | 0.925 | 7.07% | 32.212 | 0.929 | 7.07% | 32.021 | 7.33% | 32.822 |
|  |  | 4 | 0.932 | 0.931 | 7.01% | 30.879 | 0.935 | 7.01% | 30.667 | 7.334% | 31.488 |
|  | Carotenoid | 1 | 0.807 | 0.807 | 9.55% | 8.409 | 0.812 | 9.54% | 8.387 | 9.77% | 8.447 |
|  |  | 2 | 0.838 | 0.837 | 8.60% | 7.711 | 0.847 | 8.60% | 7.681 | 8.50% | 7.770 |
|  |  | 3 | 0.851 | 0.850 | 8.18% | 7.411 | 0.854 | 8.15% | 7.363 | 8.83% | 7.750 |
|  |  | 4 | 0.865 | 0.863 | 7.83% | 7.069 | 0.866 | 7.81% | 7.023 | 8.10% | 7.158 |
| Heading stage | Chlorophyll a | 1 | 0.699 | 0.697 | 11.00% | 36.987 | 0.708 | 10.98% | 36.706 | 11.23% | 37.078 |
|  |  | 2 | 0.777 | 0.774 | 8.56% | 31.979 | 0.784 | 8.51% | 31.539 | 8.86% | 33.032 |
|  |  | 3 | 0.816 | 0.812 | 7.86% | 29.141 | 0.823 | 7.83% | 28.575 | 8.18% | 30.745 |
|  |  | 4 | 0.838 | 0.833 | 7.62% | 27.467 | 0.836 | 7.58% | 26.795 | 7.92% | 30.087 |
|  | Chlorophyll b | 1 | 0.711 | 0.709 | 11.17% | 10.854 | 0.713 | 11.16% | 10.775 | 11.21% | 10.826 |
|  |  | 2 | 0.760 | 0.757 | 10.68% | 9.925 | 0.762 | 10.65% | 9.814 | 10.78% | 9.944 |
|  |  | 3 | 0.800 | 0.795 | 9.76% | 9.105 | 0.807 | 9.74% | 8.967 | 9.98% | 9.155 |
|  |  | 4 | 0.836 | 0.831 | 8.83% | 8.265 | 0.836 | 8.80% | 8.096 | 8.98% | 8.520 |
|  | Total chlorophyll | 1 | 0.708 | 0.706 | 8.52% | 47.042 | 0.711 | 9.12% | 46.615 | 9.74% | 48.188 |
|  |  | 2 | 0.782 | 0.779 | 7.73% | 40.783 | 0.791 | 8.50% | 40.293 | 8.84% | 41.337 |
|  |  | 3 | 0.821 | 0.817 | 7.15% | 37.111 | 0.827 | 7.71% | 36.545 | 7.95% | 37.468 |
|  |  | 4 | 0.856 | 0.852 | 7.01% | 33.429 | 0.859 | 7.01% | 32.782 | 7.03% | 33.956 |
|  | Carotenoid | 1 | 0.612 | 0.610 | 11.93% | 9.091 | 0.625 | 11.92% | 9.016 | 12.13% | 9.197 |
|  |  | 2 | 0.672 | 0.667 | 10.12% | 8.392 | 0.680 | 10.09% | 8.294 | 10.58% | 8.454 |
|  |  | 3 | 0.747 | 0.742 | 9.14% | 7.393 | 0.751 | 9.13% | 7.28 | 9.14% | 7.503 |
|  |  | 4 | 0.776 | 0.770 | 8.30% | 6.979 | 0.782 | 8.29% | 6.833 | 8.83% | 7.147 |
